# Highly Durable Nanoporous Cu_2–*x*_S Films for Efficient Hydrogen Evolution Electrocatalysis under Mild pH Conditions

**DOI:** 10.1021/acscatal.3c01673

**Published:** 2023-07-26

**Authors:** Roser Fernández-Climent, Jesús Redondo, Miguel García-Tecedor, Maria Chiara Spadaro, Junnan Li, Daniel Chartrand, Frederik Schiller, Jhon Pazos, Mikel F. Hurtado, Victor de la Peña O’Shea, Nikolay Kornienko, Jordi Arbiol, Sara Barja, Camilo A. Mesa, Sixto Giménez

**Affiliations:** † Institute of Advanced Materials (INAM), Universitat Jaume I, Av. de Vicente Sos Baynat, s/n, 12006 Castelló, Spain; ∥ Department of Polymers and Advanced Materials, Centro de Física de Materiales, University of the Basque Country UPV/EHU, 20018 San Sebastián, Spain; ¶ Department of Surface and Plasma Science, Faculty of Mathematics and Physics, Charles University, 180 00 Prague 8, Czech Republic; β Photoactivated Processes Unit, IMDEA Energy Institute, Parque Tecnológico de Móstoles, Avda. Ramón de la Sagra 3, 28935 Móstoles, Madrid, Spain; ‡ Catalan Institute of Nanoscience and Nanotechnology (ICN2) and BIST Campus UAB, Bellaterra 08193, Barcelona, Catalonia, Spain; ⊥ Department of Chemistry, Université de Montréal, 1375 Ave. Thérèse-Lavoie-Roux, Montréal, QC H2V 0B3, Canada; π Centro de Física de Materiales and Material Physics Center CSIC/UPV-EHU, Manuel Lardizabal 5, 20018 San Sebastián, Spain; θ Donostia International Physics Center, 20018 San Sebastián, Spain; φ IKERBASQUE, Basque Foundation for Science, 48009 Bilbao, Spain; § Research Cluster on Converging Sciences and Technologies (NBIC), Departamento de Ingeniería Electrónica, Universidad Central, Calle 5 No 21-38, Bogotá 110311, Colombia; Δ Materials Chemistry Area, Civil Engineering Department, Corporación Universitaria Minuto de Dios, Calle 80, Main Sede Bogotá, Colombia. − Nanotechnology Applications Area, Environmental Engineering Department, Universidad Militar Nueva Granada, Km 2 via Cajicá, Zipaquirá 110311, Colombia; £ ICREA, Pg. Lluís Companys 23, 08010 Barcelona, Catalonia, Spain

**Keywords:** green hydrogen, hydrogen evolution reaction, electrocatalysis, Cu-based electrodes, *operando* ECSA increase, mechanistic analysis

## Abstract

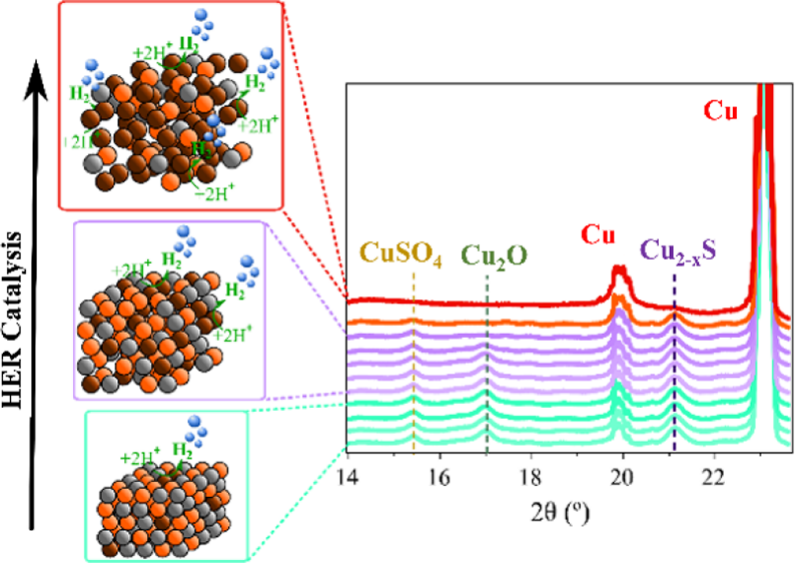

Copper-based hydrogen evolution electrocatalysts are promising materials to scale-up hydrogen production due to their reported high current densities; however, electrode durability remains a challenge. Here, we report a facile, cost-effective, and scalable synthetic route to produce Cu_2–*x*_S electrocatalysts, exhibiting hydrogen evolution rates that increase for ∼1 month of operation. Our Cu_2–*x*_S electrodes reach a state-of-the-art performance of ∼400 mA cm^–2^ at −1 V vs RHE under mild conditions (pH 8.6), with almost 100% Faradaic efficiency for hydrogen evolution. The rise in current density was found to scale with the electrode electrochemically active surface area. The increased performance of our Cu_2–*x*_S electrodes correlates with a decrease in the Tafel slope, while analyses by X-ray photoemission spectroscopy, *operando* X-ray diffraction, and *in situ* spectroelectrochemistry cooperatively revealed the Cu-centered nature of the catalytically active species. These results allowed us to increase fundamental understanding of heterogeneous electrocatalyst transformation and consequent structure–activity relationship. This facile synthesis of highly durable and efficient Cu_2–*x*_S electrocatalysts enables the development of competitive electrodes for hydrogen evolution under mild pH conditions.

## 1 Introduction

Global energy demand is expected to rise around 30% by 2040 according to the International Energy Agency (IEA).^1^ Transforming renewable electricity into green hydrogen (H_2_), *via* the water splitting process, has emerged as a promising energy vector to respond to this increasing energy demand and to decarbonize transportation, heating, and fine chemicals sectors.^2,3^ Current technologies to split water use extreme pH conditions causing environmental pollution and handling hazards. Particularly, Pt-based cathodes, which remain the state-of-the-art catalyst for the hydrogen evolution reaction (HER), are only operative in acidic media among other limitations such as scarcity and high costs.^4^ Thus, scaling-up the HER toward the gigawatt-scale requires highly efficient and durable electrocatalysts produced from earth-abundant materials and with low-cost, environmental friendly manufacturing techniques, and being operative under mild pH conditions.

In this context, electrocatalysts based on non-critical materials are attracting increasing attention to replace Pt and Ru to scale-up green H_2_ production.^5−14^ In particular, Cu chalcogenide and oxide electrocatalysts are interesting due to their remarkably low overpotentials (η) and stability compared to other transition metal catalysts toward HER.^15−17^ Outstanding η values, as low as ∼50 mV at 10 mA cm^–2^, have been reported when Cu chalcogenides are heterostructured and tested under extreme pH conditions (see Table S1 for electrode benchmarking).^18^ To reduce manufacturing costs and to simplify electrode architectures, non-heterostructured Cu_2_S electrodes are appealing to optimize. For example, Baht and Nagaraja reported a η ∼330 mV at 10 mA cm^–2^ for a hydrothermally grown Cu_2_S cathode in 1 M KOH and η ∼312 mV at 10 mA cm^–2^ when using 0.5 M H_2_SO_4_.^19^ However, these Cu_2_S electrocatalysts are measured under extreme pH conditions, *i.e.*, highly acidic or alkaline electrolytes, thus increasing the maintenance costs and the potential risks of environmental pollution when scaling-up H_2_ production. Consequently, efficient HER under mild pH conditions is key to achieve a clean and low-risk energy transition through both water and seawater splitting technologies. However, scarce studies of efficient HER on Cu_2_S electrodes in close to neutral conditions are reported.

Scaling-up green H_2_ production does not only require efficient electrodes but also require durable electrodes. Continuous operation reported for Cu-based electrocatalysts ranges from a few hours up to several days.^12,18−27^ To the best of our knowledge, the most durable Cu_2_S-containing electrocatalyst was reported by Bae *et al.*, with a Cu_2_S-Mo_2_S electrode in continuous operation for 10 days in 1 M H_2_SO_4_.^18^ However, non-heterostructured Cu_2_S electrodes measured in a mild electrolyte (1 M phosphate buffer, pH ∼7) have been measured only for 10 h.^28^ It is therefore apparent that the development of platinum group metal (PGM)-free, low-cost, and highly efficient HER electrocatalysts still remains a challenge.

**Figure 1:**
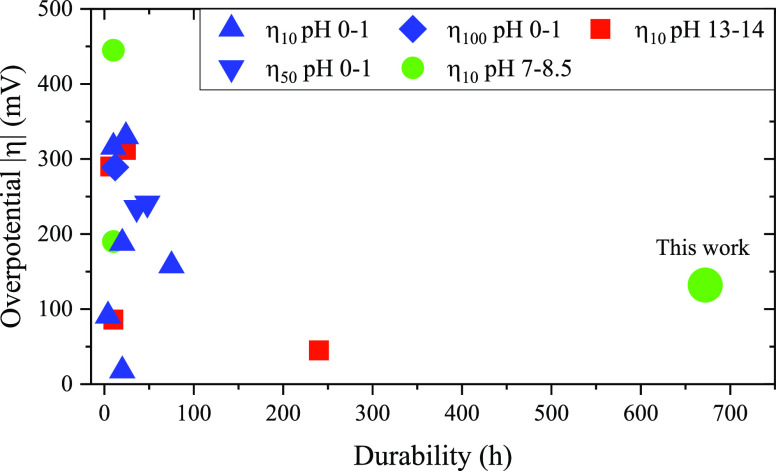
Comparative overpotentials at 10 mA cm^–2^ (η_10_), 50 mA cm^–2^ (η_50_), and 100 mA cm^–2^ (η_100_), durability time benchmark for copper sulfide electrocatalysts in Table S1, and our Cu_2–*x*_S cathode in this present work for acidic,^12,18,19,30,31^ neutral,^28,32,33^ and basic media.^19−22,26,27,34−36^

In this study, we report a cost-effective, facile method to synthetize a Cu_2–*x*_S electrocatalyst with η values as low as 130 mV at 10 mA cm^–2^ and 332 mV at 50 mA cm^–2^ under mild conditions (pH 8.6). These values compare to the lowest reported η for Cu_2–*x*_S-derived catalysts operating at extreme pH, preserving Faradaic efficiencies (FE) near 100% for HER during the tested time. Strikingly, our Cu_2–*x*_S electrocatalyst exhibits a remarkable durability of almost 1 month, which, to the best of our knowledge, is the longest reported to date for this material at such high current densities. This new benchmark durability achieved is superior compared to that reported for highly competitive noble metal electrodes, such as RuP nanoparticles (∼200 h, *i.e.*, over a week of operation),^29^ setting a technical breakthrough and a great step toward scalability for industrial renewable electrocatalytic hydrogen production. Several copper sulfide-derived electrocatalysts previously reported and collected in Table S1 are compared in terms of performance (overpotential) and durability in Figure 1. Crucially, we carried out a comprehensive structural and chemical characterization of the electrodes before, during, and after the HER test, taking advantage of the combination of *operando* spectroelectrochemistry (SEC), X-ray diffraction (XRD), and X-ray photoemission spectroscopy (XPS). Analysis of Cu_2–*x*_S electrodes simultaneously to the electrochemical tests unveils for the first time that the catalytic activity is mainly driven by catalytic centers located at the Cu species at the surface.

## 2 Results and Discussion

### 2.1 Synthesis and Structural Characterization

The synthesis of Cu_2–*x*_S was carried out on a copper substrate upon modification of a procedure reported elsewhere.^37,38^ First, a copper foil substrate was cleaned and electropolished (Figure S1). The pre-treated copper substrate was drop-casted with a Na_2_S_4_ solution forming a homogeneous layer of copper sulfide (Cu_2–*x*_S) on the substrate’s surface followed by abundant rinsing with deionized H_2_O. The catalyst mass loading was estimated as ∼0.400 mg cm^–2^. A more detailed description of the synthesis can be found in the Experimental Section.

**Figure 2:**
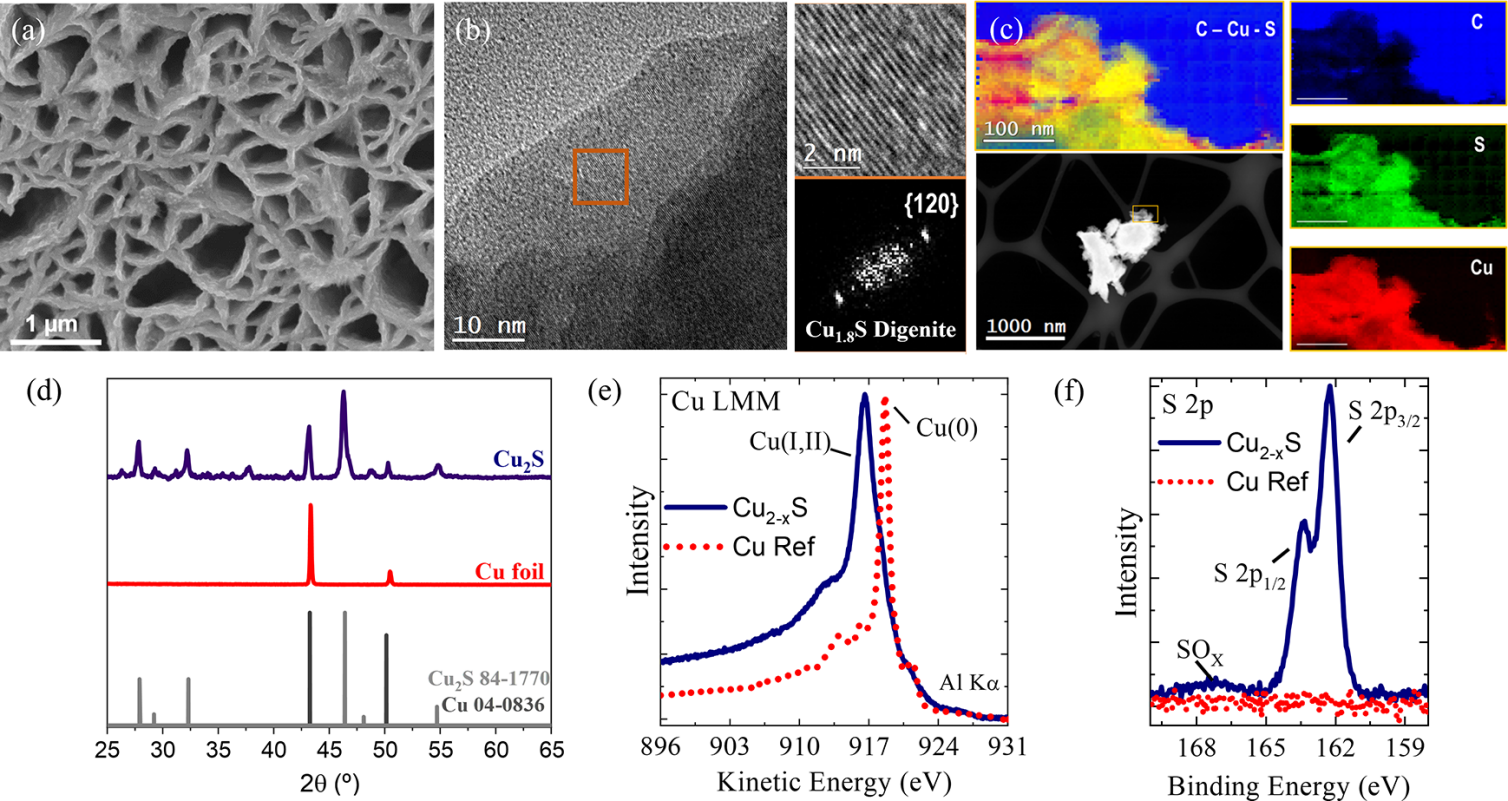
Structural characterization of the Cu_2–*x*_S electrodes. (a) Top-view SEM image, (b) HRTEM image together with the indexed power spectrum and a detailed image of the Cu_2–*x*_S structure, and (c) STEM-EELS analysis (bottom left). High-angle annular dark-field (HAADF) STEM general image of the nanostructured Cu_1.8_S (top left). EELS compositional maps showing the different elemental distributions: C (blue), S (green), and Cu (red) (right). Individual elemental maps for C (blue), S (green), and Cu (red). (d) XRD reference patterns for Cu_1.8_S (light gray) and Cu (black) and diffractograms for the electropolished copper foil (red) before the synthesis and the as-synthesized Cu_2–*x*_S on the copper substrate (blue). (e) XPS analysis for Cu LMM and (f) S 2p spectra of the Cu_2–*x*_S electrodes. Reference spectra measured on a metallic Cu substrate are shown in red dotted lines.

The morphology and structure of the as-synthesized electrodes were investigated taking advantage of different characterization techniques. Figure 2a as well as Figures S2 and S3 show scanning electron microscopy (SEM), atomic force microscopy images (AFM), and energy-dispersive spectroscopy (EDS) chemical composition (Figure S2b) of the Cu_2–*x*_S film exhibiting a thickness of ∼6 μm (Figure S2c,d) featuring a highly porous structure characteristic of foamy materials, in agreement with previous reports.^39,40^ This is in contrast to the flat surface of electropolished copper foil before the synthesis, as shown in Figure S2e. The EDS analysis of the film confirms the composition and stoichiometry of Cu_2_S (Figure S2b and Table S2). High-resolution transmission electron microscopy (HRTEM) analysis reveals a high-temperature Cu_1.8_S digenite structure (space group, s.g. 224) where the family of (211) planes could be identified, from a measured interplanar distance of 0.243 nm (HRTEM image and FFT analysis in Figure 2b). Additionally, the chemical composition of the electrodes was confirmed by electron energy loss spectroscopy (EELS) in scanning transmission electron microscopy (STEM) mode. STEM-EELS spectrum image composition maps are shown in Figure 2c. In particular, the S L-edge at 165 eV (green), C K-edge at 284 eV (blue), and Cu L-edge at 931 eV (red) were used. Note that C is only present in the TEM grid and not in the electrocatalyst. Furthermore, XRD of the as-synthesized Cu_2−*x*_S electrocatalyst showed peaks at 2θ values of 27.84, 32.23, 46.23, and 54.9° that could be indexed to (111), (200), (220), and (311) planes of the Cu_1.8_S cubic phase, respectively (PDF no. 24-61).^41,42^ Two additional minor peaks are observed at 2θ values of 36.5 and 42.4° and indexed to the (111) and (200) crystal planes, respectively (JCPDS no. 05-0667).^43^ These peaks are assigned to the formation of superficial Cu_2_O after the synthesis. The characteristic peaks for metallic Cu (PDF #04-0836) are also visible in the diffractogram, with a preferred orientation (111) (Figure 2d). Finally, UV–vis–NIR characterization shows a broad absorption spectrum up to ∼1300 nm and a second NIR band that is tentatively assigned to a localized surface plasmon resonance due to the Cu deficiency in the material (Figure S2).^44,45^

Chemical analysis of the surface of the Cu_2–*x*_S electrode was carried out by XPS. Figure 2e and Figure 2f show the representative Cu LMM and S 2p spectra of the Cu_2–*x*_S electrodes (blue solid line), respectively. The reference spectra of metallic Cu are shown in red dotted line. The surface of the electrodes was gently sputtered with Ar^+^ ions in the analysis chamber prior to the XPS characterization to remove adsorbed species from air exposure after the synthesis (see the Experimental Section and Figure S4). The XPS data unveils a Cu_2−*x*_S stoichiometry of the electrodes.^46^ The Cu LMM Auger emission lineshape (Figure 2e) is compatible with the presence of Cu(I) or Cu(II) chemical states,^47^ with the absence of metallic Cu. The Cu 2p spectral region of the fresh electrode (Figure S4) reveals a well-defined 2p_2/3_ component with a binding energy at 932.7 eV. The absence of satellite peaks at ∼943.6 eV allows us to exclude the presence of Cu(II) species.^47,48^ The S 2p spectrum (Figure 2f) shows a strong doublet peak with a S 2p_3/2_ binding energy at 162.2 eV. The observed binding energy is attributed to the S^2–^ species bonded with Cu^+^ to form Cu_2−*x*_S. The presence of a broader and less intense feature at 167 eV may be caused by the presence of residual SO_*x*_ species on the catalyst surface oxidized in air.

### 2.2 Electrocatalytic Performance

**Figure 3:**
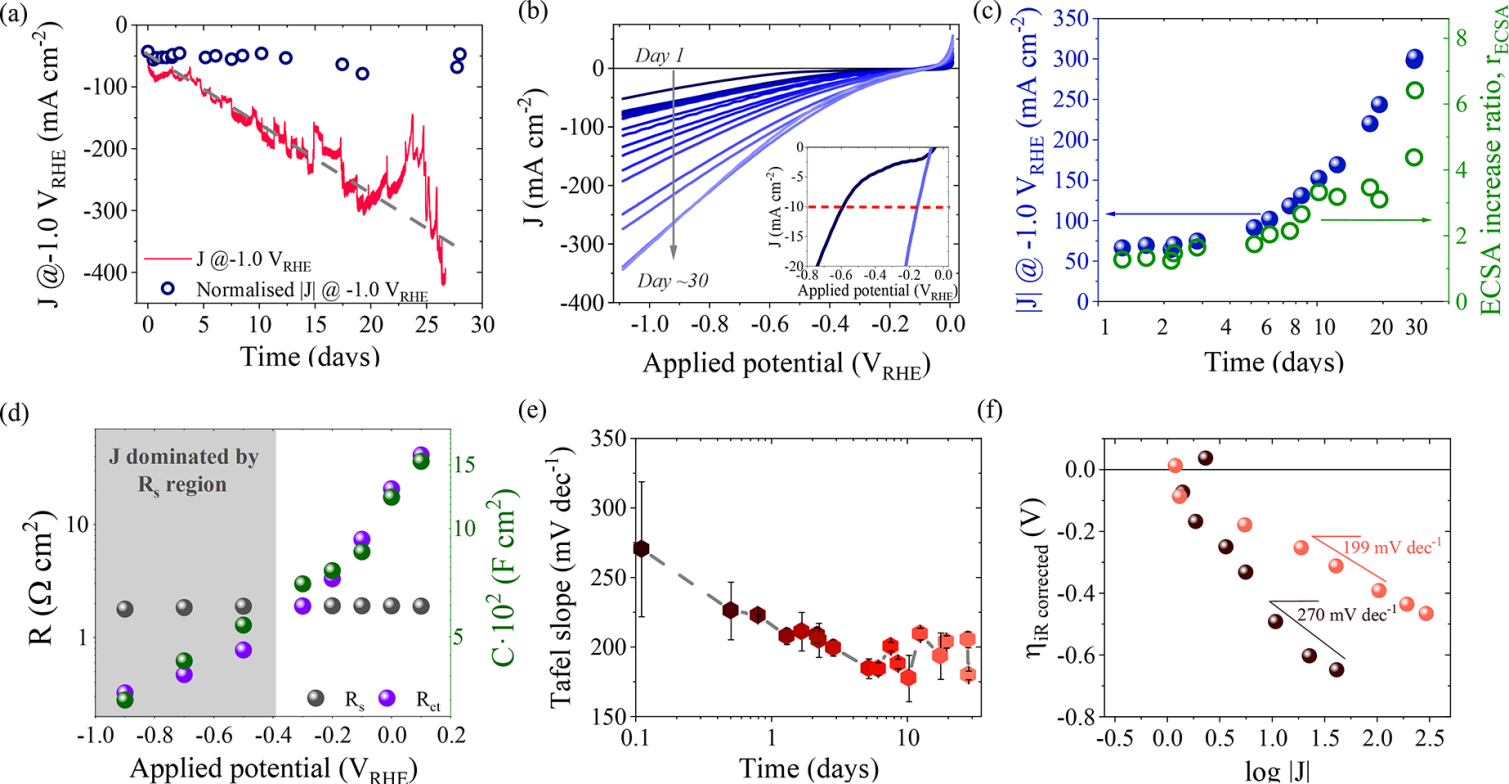
Electrocatalytic performance characterization of the Cu_2–*x*_S electrodes. (a) Chronoamperometric measurement at −1 V vs RHE of the Cu_2–*x*_S catalyst for 28 days of continuous operation. The dashed gray line represents the quasi-linear increase in the catalytic current density as a function of operation time. Steady-state currents at −1.0 V vs RHE normalized by the electrochemical surface area (ECSA) are shown as light blue empty dots. (b) Linear sweep voltammograms (LSV), measured at 20 mV s^–1^, of the same Cu_2–*x*_S electrode as a function of operation time between day 1, *i.e.*, freshly synthesized catalyst (darker blue), and after 28 days (lighter blue) of continuous operation. Inset: zoom between the first and the last LSV to compare the overpotential at −10 mA cm^–2^ (dashed red line). (c) Cathodic current densities (|*J*|) measured at −1.0 V vs RHE, from panel (b) (blue filled dots) compared to the ECSA increase ratio (empty green dots, *R*_ECSA_) calculated using eq 1. Note that the time is in the log scale. (d) Series (*R*_S_) and charge transfer (*R*_CT_) resistances and capacitance, *R*_S_ (gray dots), *R*_CT_ (violet dots), and C (green dots) at the 28th day of measurement. The gray area denotes the potential region where *R*_CT_ < *R*_S_. (e) Tafel plot comparing the 1st (dark red data) with the 28th (pink data) day of measurement of the steady-state catalytic currents (see Figure S14 for the corresponding steady-state LSVs) and (f) Tafel slope values as a function of operation time obtained from panel (e). All measurements were performed in 0.1 M KHCO_3_ (pH 8.6).

The functional evaluation of our Cu_2–*x*_S electrocatalyst for H_2_ evolution was assessed by chronoamperometric (CA) measurements performed at −1 V vs RHE and simultaneous measurements of the FE. Remarkably, our Cu_2–*x*_S electrocatalyst exhibits a record-breaking durability of ∼1 month of continuous operation in a three-electrode configuration (Figure 3a). To ensure that the catalyst is highly efficient to evolve hydrogen, not only at the beginning but also during days of continuous operation, subsequent gas chromatography (GC) measurements were carried out to verify that the FE remains close to 100% over time (Figure S5). This striking durability outperforms the best Cu_2–*x*_S and Cu_2–*x*_S-derived electrocatalyst reported to date by nine times^20^ (see Figure 1 and Table S1 for reported durability benchmark) as well as highly competent noble metal electrodes, such as RuP nanoparticles (∼200 h, *i.e.*, over a week of operation).^29^ Strikingly, the current density (*J*), at −1 V vs RHE, exhibited by our Cu_2–*x*_S electrocatalyst increases monotonically by 8-fold, from approximately −50 to approximately −400 mA cm^–2^ (Figure 3a), during the operation period. Along the durability test, the CA measurement was systematically interrupted to investigate the nature of the increase in *J* by performing complementary electrochemical measurements. Linear sweep voltammetries (LSVs) in Figure 3b show the increasing *J*, at matched potentials, with time as indicated by the gray arrow, following the increase observed in the CA (Figure 3a). This enhanced performance was also observed by an anodic shift in the overpotential to reach −10 mA·cm^–2^ (geometric area), from approximately −530 mV to a benchmark value of approximately −130 mV (Figure 3b, inset). It is apparent from these results that our Cu_2–*x*_S electrode requires ∼60 mV less overpotential compared to a previously reported Cu_2–*x*_S cathode for HER in the KPi electrolyte at pH 7.^28^ Blank LSVs performed on the bare Cu substrate in comparison with the Cu_2–*x*_S electrocatalyst (Figure S6) do not reveal a significant HER activity, clearly showing that the HER catalytic activity of the electrode arises from the Cu_2–*x*_S material. Furthermore, the here investigated Cu_2–*x*_S electrodes were tested in a two-electrode configuration reaching ∼15 mA cm^–2^ with a remarkable durability of 65 h (Figure S7). Compared to other Cu_2−*x*_S and Cu_2−*x*_S-derived electrocatalysts, our electrode exhibits among the lowest overpotential values obtained to date for electrocatalysts based on Cu_2–*x*_S (Figure 1 and Table S1 for a state-of-the-art comparison).^19,49^ Note that most of the works included in Table S1 were tested under extreme pH conditions (highly acidic or alkaline), in which faster reaction kinetics are expected. However, our motivation herein is to focus on moderate pH values to overcome the problems derived from the use of highly acidic or alkaline media accelerating the implementation of both neutral and seawater splitting technologies.

To rationalize such an increasing performance of our Cu_2-*x*_S electrodes shown in Figure 3a,b and considering how the electrode surface increases during electrochemistry (Figure S8), we propose that an *operando* increase in the surface area is taking place. A larger surface area can facilitate the exposure of more active sites improving the catalytic activity for HER while minimizing mass transport limitations.^20,50,51^ To test this hypothesis, the double-layer capacitance of the Cu_2–*x*_S catalyst was monitored systematically by electrochemical impedance spectroscopy (EIS) during the whole operation time. Figure 3c shows that the ratio of the electrochemical surface area increase (*r*_ECSA_) in our Cu_2–*x*_S catalyst was determined in eq 1.
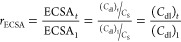
1where ECSA_1_ and ECSA_*t*_ correspond to the electrochemical surface area (ECSA), as reported elsewhere,^52^ of day 1 (ECSA_1_) and that of subsequent measurements as a function of operation time (ECSA_*t*_). *C*_s_ is the specific capacitance, which is canceled in eq 1, and similarly to the ECSA, (*C*_dl_)_1_ and (*C*_dl_)_*t*_ correspond to the double layer capacitance of the first day (*C*_dl_)_1_ and that of subsequent measurements (*C*_dl_)_*t*_. These *C*_dl_ data were obtained from the low-frequency capacitance plateau region (0.5–10 Hz), *i.e.*, the Bode plot in Figure S9, as previously reported*.*^51,53^

Figure 3c compares *J* (blue filled dots) with the increase in electrochemical surface area (*r*_ECSA_, see eq 1, green empty dots). It is apparent from Figure 3c that *r*_ECSA_ increases by a factor of ∼6.5 times from day 1 to day 28 of continuous operation. We further use *r*_ECSA_ to normalize the LSVs from Figure 3b (see Figure S10). Upon normalization, *J* at −1 vs RHE appears to be almost invariant as a function of time (Figure 3a, blue empty dots). The observed cathodic increase in *J* can be mostly attributed to a higher number of exposed active sites as evidenced by the increase in ECSA measured by EIS. This surface area increase allows our catalyst to reach remarkable current densities while preserving the FE toward H_2_. Interestingly, a similar increased HER performance was reported by Yang and co-workers on CoMnS-based electrodes, where the increased current density was associated with an ECSA increase due to a partial crystallinity loss of the electrocatalyst.^54^ Concomitant with the noticeable visual increase in the surface area of our Cu_2–*x*_S electrocatalyst during the HER (Figure S8 and Video S1), in Section 2.3, we also discuss possible cathodic corrosion processes contributing to the observed ECSA increase.

We now turn to analyze the charge transfer mechanism by means of EIS, for which we selected the set of data of day 28, *i.e.*, at the highest current density reported here (approximately −400 mA cm^–2^; Figure 3b). Figure 3d shows the evolution of both, series (*R*_S_) and charge transfer (*R*_CT_), resistances, fitted to Randle’s circuit (see the Supporting Information for further discussion and Nyquist plots; Figure S11) with applied bias. The crossover point where *R*_CT_ becomes smaller than *R*_S_ is observed at approximately −0.3 V vs RHE in Figure 3d coinciding with the HER catalytic onset potential (see LSVs in Figure 3b), indicating that at more cathodic potentials (gray shaded region in Figure 3d), the delivered current is limited by the device design and engineering (reflected by *R*_S_), rather than by surface electrocatalysis (controlled by *R*_CT_). On the other hand, *R*_S_ exhibits a constant behavior with bias but decreases as a function of the operation time due to the increase in the ionic concentration of the solution (Figure S12),^55,56^ after ∼1 month of constantly adding electrolyte solution to the employed static electrochemical cell. Furthermore, although a small local pH variation (basification of 0.5–1 pH units at distance to the cathode <1 cm) was detected, the pH of the bulk electrolyte was stable along the measurement. Interestingly, the extracted capacitances as a function of increasing the negative bias shown in Figure 3d (green dots) exhibit a constant decreasing trend. This decrease in the capacitance at the catalytic region could partially be related to the evolution of H_2_ gas since the produced bubbles can decrease the electrochemical surface area, although it could also be attributed to a decrease in the accumulated species at the catalyst/electrolyte interface during the catalysis when the charge transfer process is enhanced, as previously reported for water oxidation electrocatalysts.^57^Figure S13 also shows higher extracted resistances and capacitances for the measurements at lower currents, *i.e.*, between day 1 and 28, evidencing the enhanced charge transfer kinetics concomitant to decreased charge accumulation with operation time.

Tafel analysis was performed to gain insights into the charge transfer HER kinetics. Steady-state LSVs (Figure S14) were performed concomitant to ECSA determination to determine the iR drop corrected Tafel slopes (Figure 3e,f).^58^ Initially, the electrocatalyst exhibits a Tafel slope of 270 mV dec^–1^, which gradually decreases during the first week of plateauing around ∼190 mV dec^–1^ until ∼1 month, indicating that the HER kinetics of the Cu_2–*x*_S is faster after 1 month of continuous operation correlating with the increase in *J* and the decrease in η (Figure 3b and inset, respectively) as well as with the decrease in *R*_CT_ (Figure S13a). Note that by the time the Tafel slope becomes approximately constant in Figure 3e, an inflection point in the currents and the ECSA ratio occurs and higher *J* values and ECSA ratios are observed (Figure 3c).^59−61^ Overall, the evolution of both ECSA and Tafel slopes suggests a rather constant intrinsic activity of our Cu_2–*x*_S catalyst.

### 2.3 HER Mechanistic Analysis

The evolution of the structure and composition of the Cu_2–*x*_S electrodes during the HER process have been studied by combining a set of complementary *operando* and post-mortem analysis techniques.

**Figure 4:**
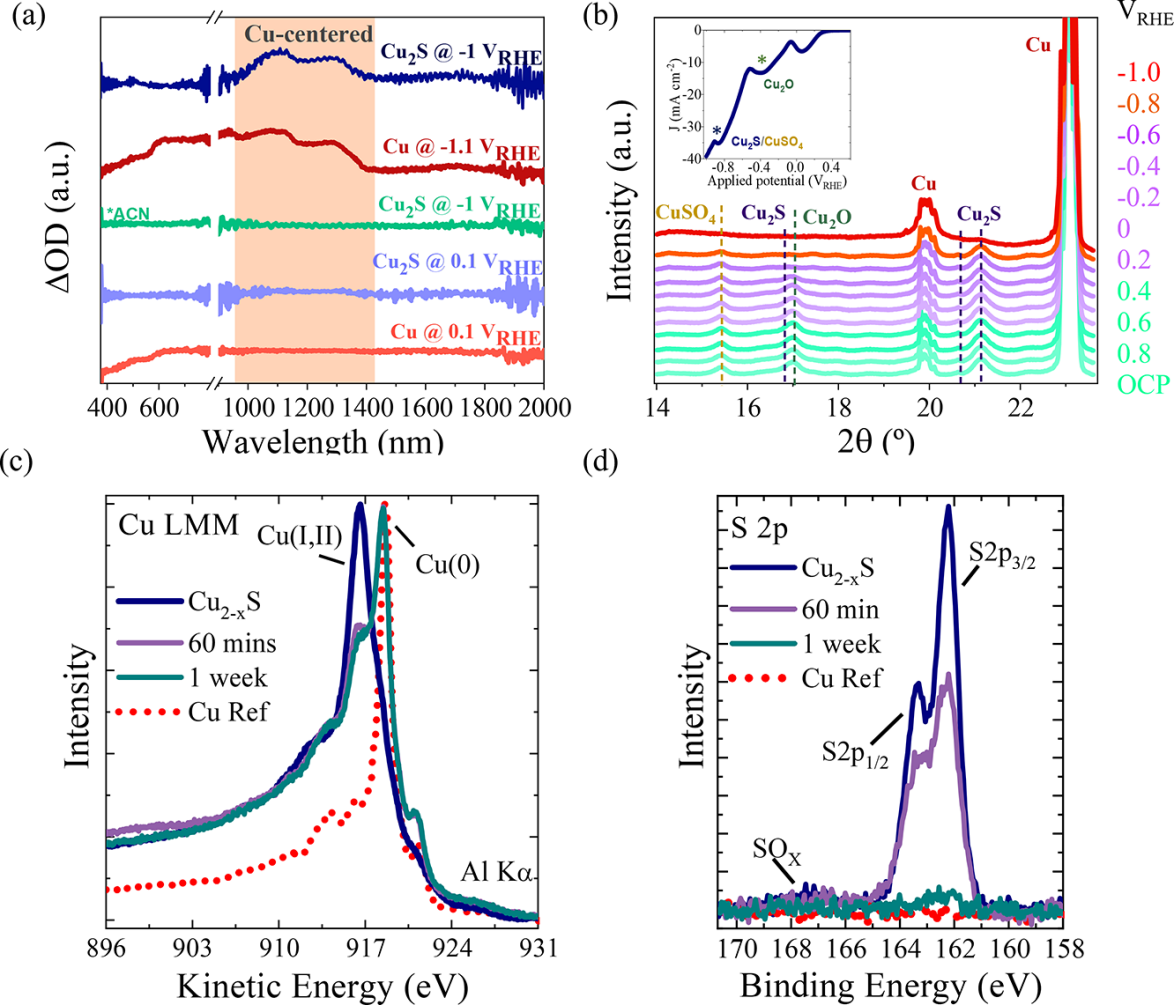
*Operando* and under inert atmosphere characterization. (a) Differential optical density spectra of the Cu_2–*x*_S (light and dark blue) and reference Cu foil (light and dark red) electrodes as a function of potential. For reference, Cu_2–*x*_S differential spectra were measured also in 0.1 M TBAP in acetonitrile, see further analysis and peak assignment in Figure S15. (b) *Operando* XRD diffractograms at different potentials from OCP to −1.0 V vs RHE. Inset: LSV; (c, d) reference and post electrochemical XPS measurements for (c) Cu LMM and (d) S 2p spectra.

First, we performed *operando* spectroelectrochemical (SEC) analysis,^62^ where the UV–vis–NIR absorption spectra of our Cu_2–*x*_S electrode were monitored while holding the electrode at different potentials. Figure 4a compares the differential spectral fingerprint (in the UV–vis–NIR range) of the Cu_2–*x*_S electrode as a function of the applied potential (see Figures S15 and S16 for the full set of spectra). The counterpart spectra of the bare Cu foil are represented as reference. The relatively flat spectra (light green and blue data) for both Cu_2–*x*_S and Cu electrodes are obtained at low potentials (0.1 V vs RHE) where the current density is close to zero (Figure 3b). These flat optical fingerprints evolve to spectra with at least two peaks at ∼1080 and ∼1270 nm when applying higher potentials where HER catalytic currents take place.^63−65^ With the purpose of assigning the observed peaks, blank measurements were also performed in an inert electrolyte where hydrogen evolution is not expected. When the Cu_2–*x*_S electrode is under equivalent applied potentials in acetonitrile (Figure 4a, dark red spectrum), these two peaks are not present. This observation suggests that the signals observed at ∼1080 and ∼1270 nm correspond to protons bound to both Cu_2–*x*_S and electropolished Cu surfaces. The development of this optical fingerprint in the catalytic region allows us to assign the peaks at ∼1080 and ∼1270 nm to species accumulating at the rate-determining step of the HER. Note that these signals are only observed, while HER is taking place and is equivalent between both the Cu_2–*x*_S catalyst and bare Cu foil substrate (see the shaded region in Figure 4a). This parallelism between both Cu_2–*x*_S and bare Cu electrodes suggests that the HER catalytic activity of Cu_2–*x*_S is driven by centers located at Cu species at the Cu_2–*x*_S surface. To further assign each optical signal observed in the spectra, additional experiments were performed by adding 10% volume of water to the inert electrolyte. Figure S15 in SEC supplementary analysis shows the UV–vis–NIR absorption spectra of Cu_2–*x*_S electrocatalyst measured in 0.1 M KHCO_3_ and 0.1 M TBAP in acetonitrile and as reference a bare Cu electrode in 0.1 M KHCO_3_ under a strong negative bias. Optical signals at ∼550 and 1370 nm are assigned to optical absorptions from the Cu_2–*x*_S electrode in agreement with previous reports.^45,63^ Additionally, a NIR absorption band from free H_2_O (*i.e.*, water molecules in the solvent not interacting with the catalyst surface) is also observed at ∼1500 nm.^66^ Interestingly, an optical absorption at ∼1200 nm emerges when the bias increases toward more negative potentials and it is discussed in the Supporting Information.

*Operando* XRD analysis was carried out on our Cu_2–*x*_S electrocatalyst (Figure 4b) to unveil crystallinity changes taking place under catalytic conditions. Figure 4b shows the evolution of the XRD patterns when the potential is progressively increased from open circuit potential (OCP, *i.e.*, ∼0.2 V vs RHE) to −1 V vs RHE. Under OCP conditions, characteristic phases of Cu, Cu_2_O, Cu_1.8_S, and CuSO_4_ are observed. The Cu_2_O phase disappeared between −0.4 and −0.6 V vs RHE (green asterisk in the cathodic linear sweep voltammogram, inset in Figure 4b). At more cathodic potentials (−0.8 and −1 V vs RHE), the signals related to the Cu_1.8_S and the secondary CuSO_4_ phase also vanished, related to the following cathodic wave (blue asterisk in the inset of Figure 4b) in the linear sweep voltammogram and the corresponding growth of the Cu signal around −1.0 V vs RHE. A more precise quantification of the evolution of the Cu peak was elusive since the Cu foil underneath results in the oscillation in intensity.

Complementary, XPS measurements were performed to probe the chemical state and composition of the surface of the Cu_2–*x*_S electrodes immediately after HER. The electrochemical experiments and emersion of the sample were carried out in a glovebox under a N_2_ (99.999%) inert atmosphere. Then, the sample transfer to the XPS setup was conducted using a portable glovebox under N_2_, which was adapted to be directly attached to the ultra-high vacuum (UHV, *P* < 10^–9^ mbar) system. Copper sulfide compounds are known to readily oxidize upon air exposure.^67,68^ Transfer and post-mortem characterization in air could mislead the interpretation of the actual composition of the surface (see Figure S17 for a comparison of air vs N_2_ exposed samples). We also notice the relevance of an abundant rinsing of the electrode after HER to avoid residues of the bulk electrolyte solution on the sample surface, which might inhibit the photoemitted signal from the surface of the electrode (see Figure S18 for a comparison of washed versus unwashed samples). Figure 4c and Figure 4d show, respectively, the Cu LMM Auger transition and S 2p core level emission of the electrodes after 60 min (purple solid line) and 1 week (blue petroleum solid line) of operation. The spectra from the as-synthesized electrode (blue lines) and metallic Cu (red dotted lines) from Figure 2e,f are included as reference. The surface of the electrodes was gently sputtered with Ar^+^ ions prior to the XPS characterization to eliminate the remaining residues from the transfer procedure. The electrodes after 60 min and 1 week of operation have evolved to a reduced metallic state (Figure 4c), attributed to the increase in the Cu^0^ (K.E. 918.4 eV) component in the Cu LMM spectra, simultaneous to the decrease in the Cu (I and II) (K.E. 917 eV) components observed in the as-synthesized electrodes. Notably, a decrease in the S 2p component takes place with operation time (Figure 4d), observing almost complete depletion of S after 1 week of catalytic activity, in agreement with previously discussed *operando* XRD experiments. Such depletion of S could be associated with an initial formation of S vacancies, which evolves to a dissolution of S to the electrolyte, as shown in Figure S19. The S 2p XPS spectrum of the fresh Cu_2–*x*_S electrode (petrol blue line; Figure 4d) shows two peaks located at 162.2 eV (S 2p_3/2_) and 163.37 eV (S 2p_1/2_), corresponding to the spin–orbit splitting (Δ*E* = 1.17 eV) of the 2p orbital. Both components contain equivalent chemical information. The S 2p spectrum of the electrode after 60 min of operation (purple line; Figure 4d) presents a substantial (about 1/3) reduction of its initial intensity. The deconvoluted analysis of the spectral features reveals the emergence of an additional component at higher binding energies (*E*(2p_3/2_) = 162.7 eV), which adds up to approximately 38% of the total S signal (see Figure S20). We tentatively attribute this higher-energy component as the fingerprint of a transient phase at the beginning of the electrochemical process, which precludes the total depletion of S from the electrode. All fitting parameters are provided in the Supporting Information.

Further post-mortem characterization by SEM, XRD, and HRTEM after removing the electrode from the solution in air was not informative due to the formation of an extensive oxide layer (not shown).

**Figure 5:**
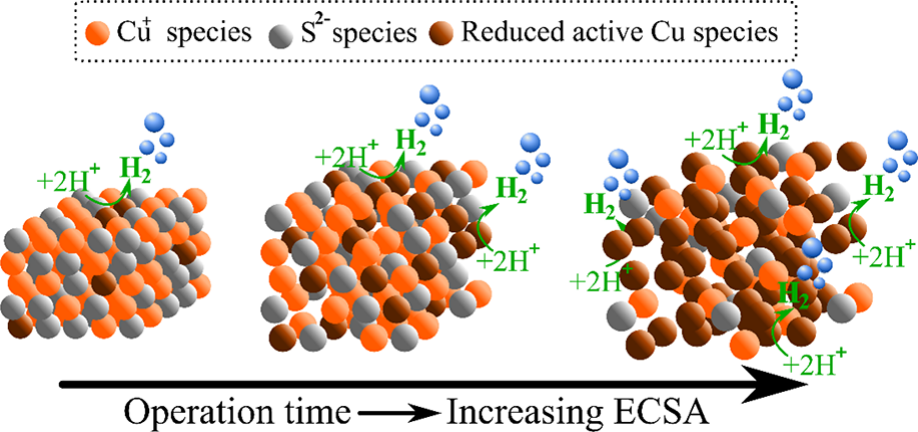
Schematic representation of the proposed changes in crystallinity of our Cu_2−*x*_S electrocatalyst, concomitant to the S (gray) leaching process that leads to more catalytically active Cu species (brown) to be exposed leading to faster HER.

In general, we observe that the increased HER performance correlates with an *operando* ECSA increase in our catalyst. As the HER proceeds, we detect severe changes in crystallinity and a progressive S leaching process leading to a highly porous and active catalytic micro/nanostructure, as shown schematically in Figure 5. The increase in porosity is consistent with an increase in the ECSA leading to a higher density of exposed active sites that are found to be centered at Cu species.

## 3 Conclusions

In summary, we report an earth-abundant Cu_2–*x*_S electrocatalyst for sustainable H_2_ production. A simple, fast, environmentally friendly, and cost-effective synthetic route is presented to obtain highly efficient Cu_2–*x*_S electrodes for H_2_ production with an FE of ∼100% and current densities that increase from 50 to ∼400 mA·cm^–2^, with remarkably low overpotentials (−130 mV at 10 mA·cm^–2^) in a mild pH electrolyte, for almost 1 month of continuous operation. All these features make our electrocatalyst very competitive for its scalability and subsequent technological deployment. We presented a comprehensive electrochemical, structural, and chemical analysis on the mechanistic pathway of the reaction and the electrode evolution under *operando* conditions. Our study unveils a direct relationship between the increase in the current densities and the ECSA and establishes a faster HER kinetics with increasing operation time. SEC analysis reported for the first time for copper-based films for HER, in situ XRD analysis, and air-free XPS characterization of the post-mortem electrode demonstrates that the catalytically active sites are located on Cu species. In conclusion, we believe that the Earth abundance of the developed Cu_2–*x*_S electrocatalyst and the fast, environmentally friendly, and cost-effective synthetic strategy, combined with the excellent performance in almost neutral pH conditions and relevant guidelines for catalyst design and optimization based on mechanistic analysis, may pave the way for the future design of highly efficient, durable, and economically competitive hydrogen evolution electrocatalysts to be used in a wide range of electrosynthetic applications.

## 4 Experimental Section

### 4.1 Electrode Preparation

#### 4.1.1 Substrate Pre-Treatment

Prior to film preparation, copper foil substrates (Goodfellow, 0.2 mm thick, 99.9%) were cleaned with soap/Milli-Q water, acetone, and ethanol. After drying the substrates under airflow, the substrates were electropolished in phosphoric acid (Alfa-Aesar, 85%) potentiostatically 1 min at 3 V and 10 min at 2 V vs Carbon GDL (FuelCellStore, Sigracet 39 BC) in a two-electrode configuration. Finally, the electropolished copper foil was rinsed with abundant deionized water and dried in air.

#### 4.1.2 Na_2_S_4_ Solution Preparation

A Na_2_S_4_ polysulfide solution was prepared following a procedure described in previous works^38^ mixing NaOH (Scharlab), Na_2_SO_4_ (Alfa Aesar, 98%), and S (Sigma-Aldrich, 99.98%) stoichiometrically with deionized (DI) water in a three-neck round bottom flask. The resulting mixture was heated at 313 K with vigorous stirring and constant purified nitrogen bubbling for 3 h. Subsequently, the temperature of the solution was increased to 373 K until a color change from yellow to black was observed.

#### 4.1.3 Electrode Synthesis

The Na_2_S_4_ solution was drop-casted onto the pre-treated copper foil substrates. Then, the electrode was rinsed with abundant DI water to remove the excess of the polysulfide solution and dried under airflow. The prepared films showed the whole surface of the substrate covered by a homogeneous black Cu_2–*x*_S thin layer.

### 4.2 Characterization Techniques

SEM measurements were performed with a JSM-7000F JEOL FEG-SEM system (Tokyo, Japan) equipped with an INCA 400 Oxford EDS analyzer (Oxford, U.K.) operating at 15 kV (see Figure 2a and Figure S2 and Table S2 for the Cu_2−*x*_S EDS analysis). High-resolution transmission electron microscopy (HRTEM) and scanning transmission electron microscopy (STEM) investigations were performed on a field emission gun FEI Tecnai F20 microscope. High-angle annular dark-field (HAADF) STEM was combined with electron energy loss spectroscopy (EELS) in a Tecnai microscope by using a GATAN QUANTUM energy filter to obtain compositional maps.

Profilometer measurements were performed on a mechanical profilometer Veeco model Dektak 6 (see Figure S2d).

X-ray diffractograms were recorded using a Rigaku Miniflex 600 (Rigaku Corporation, Tokyo, Japan) with Cu Kα radiation (λ = 1.5418 Å) (see Figure 2d).

Topographic images of the samples were measured by atomic force microscopy (AFM) using an Asylum Research Cypher ES (Oxford Instruments-Asylum Research, Santa Barbara, USA) in tapping mode under ambient conditions with a silicon cantilever AC160TSA-R3 (Olympus, Tokyo, Japan) with spring constant *k* = 25.4 N/m, resonant frequency *f* = 291 kHz, and a tip radius of 7 nm (see Figure S3).

### 4.3 X-ray Photoemission Spectroscopy Measurements

XPS has been carried out holding the sample at room temperature and illuminating it with monochromatized Al K_α_ light (*h*ν = 1486.6 eV) from a microfocus setup (SPECS Focus 600, spot size 500 μm). The excited photelectrons were collected by a SPECS 150 hemispherical analyzer at emission and incidence angles of 40 and 60°, respectively. The overall experimental resolution was extracted from Fermi edge analysis of a reference gold sample and resulted in 0.4 eV. To average inhomogeneities of the electrodes, especially after the HER, different positions of the samples were characterized by XPS. The data presented here is the result of averaging multiple spectra of different positions for each sample.

The characterized electrodes before the HER were sputtered in UHV to remove the oxide layers that form upon exposure of Cu_2–*x*_S to air^67^ and the adventitious C that adsorbs. A comparison of the Cu 2p, Cu LMM, O 1s, C 1s, and S 2p spectra before and after the sputtering can be found in Figure S4.

The data of electrodes after the HER corresponds to MiliQ-washed, N_2_ environment-produced samples. MiliQ water was used to rinse the electrode after the HER to remove electrolyte residues.^46^ High purity (99.999%) N_2_ was used to reduce unwanted oxidation from air exposure during the HER and manipulation of the samples. The samples were kept in a commercial glovebox during HER under a continuous flow of N_2_, transported in a sealed N_2_ container, and inserted in the UHV load-lock without breaking the N_2_ atmosphere. A detailed comparison of the impact of sample rinsing and transport in N_2_ can be found in Figures S17 and S18, respectively.

### 4.4 Electrochemical Measurements

The electrochemical performance of the electrodes was evaluated by linear sweep voltammetry (LSV) and chronoamperometry (CA) using an Autolab potentiostat/galvanostat PGSTAT302 in a three-electrode homemade sealed electrochemical cell suitable for gas chromatography. Aqueous Ag/AgCl (0.3 M KCl, ALS, Japan) and a Pt mesh (Alfa Aesar) were used as reference and counter electrodes, respectively, and a 1 cm^2^ geometrical area Cu_2–*x*_S film as the working electrode. Potassium bicarbonate (KHCO_3_) was chosen as the electrolyte due to its moderate pH, electrode performance (compared to the KPi electrolyte), and the potential implementation of our electrocatalysts in sea water reduction. All the potentials reported herein referred to the reversible hydrogen electrode (RHE) and were calculated through the Nernst equation *V*_RHE_ = *V*_Ag/AgCl_ + 0.199 + 0.059·pH. Data was not iR corrected unless otherwise stated. LSVs were performed between 0.1 and −1 V vs RHE with a scan rate of 0.02 V/s and step of 0.002 V, and CA was measured at a constant voltage of −1 V vs RHE with an interval of 10 s. The irregular line obtained from the chronoamperometric measurement (Figure 3a) is due to a volume change of electrolytes given the constant evaporation and reaction of it and subsequent refilling to 20 mL. Two-electrode durability tests were also carried out to evaluate the electrocatalyst performance in real operation conditions (Figure S7) since it has been reported that three- and two-electrode characterization lead to significant differences in stability.^69^ Cu_2–*x*_S was measured versus a Pt mesh at −3 V in 0.1 M KHCO_3_ for 65 h.

Electrochemical impedance spectroscopy (EIS) measurements were performed between 0.1 Hz and 0.1 MHz with 10 mV of amplitude perturbation. The EIS raw data were analyzed with ZView software (Scribner Associates), fitting the raw data to an equivalent circuit model (see Figure S11, inset) for extracting both capacitances and resistances. To investigate the dependence between the series resistance (*R*_S_) and the concentration of the electrolyte, equivalent EIS data was obtained, using fresh samples, as a function of the KHCO_3_ concentration (see Figure S12, inset).

Tafel analyses were performed by measuring, considering that the required experimental conditions for a valid analysis were met (*i.e.*, no Ohmic distortions, no background currents, and at least one decade of linearity in the Tafel curve).^70^ Since the Tafel slopes are overpotential-dependent, Tafel analysis must to be done with data acquired in a steady state and free of iR drop.^58^ Steady-state LSVs (ssLSVs) were built by measuring subsequent 5 min chronoamperometries and averaging the last 60 s of the current vs time response, at each applied potential (Figure S14). Then, the iR drop corrected overpotentials were plotted vs log |*J*| extracting from these curves the Tafel slopes (Figure 3e,f). Although the obtained Tafel slopes are higher than expected for Tafel, Heyrovsky, or Volmer mechanisms, the observed trends are still informative for the evolution of the intrinsic activity of our catalyst.

Spectroelectrochemical (SEC) analysis was performed by coupling electrochemical and spectroscopic techniques. Chronoamperometric measurements were carried out for the copper sulfide electrocatalyst and on a bare copper substrate in a three-electrode Raman electrochemical flow cell (RedoxMe) at different potentials while taking optical absorption spectra on a spectrophotometer UV–vis–NIR (Perkin Elmer 1050+). To obtain additional data, the reactions were carried out both in the bicarbonate electrolyte and in dry acetonitrile with and without adding water. Data was acquired in diffuse reflectance mode using an integrating sphere, and the data was further converted into absorbance by the Kubelka–Munk equation. The measured spectra shown in Figure 4a are presented as differential spectra (ΔOD) from the absorption data.

### 4.5 Product Determination and Quantification

The H_2_ generation was monitored by gas chromatography measurements coupling the sealed cell to an Agilent Micro-GC gas chromatograph. Chronoamperometric measurements were carried out at three different potentials (−0.5, −0.8, and −1 V vs RHE) for half an hour each. The Faradaic efficiency (FE) was estimated through the relation FE (%) = H_2_(exp)/H_2_(theo), where H_2_(exp) is the amount of evolved H_2_ in mol, monitored every 5 min, and H_2_(theo) corresponds to the theoretical H_2_ evolved calculated by Faraday’s law: *n*(mol) = *j*_H2_*t*/*nF*, where *j*_H2_ is the current density recorded during the chronoamperometry measurement, *t* is the time in seconds, *n* is the number of electrons transferred in the reaction, and *F* is the Faraday constant, 96,485.33 C mol^–1^. Furthermore, using the same procedure, long-term H_2_ Faradaic efficiency was determined by monitoring the evolved H_2_ every 6 h over 1 week of reaction (Figure S5).

Total reflection X-ray fluorescence (TXRF) analysis of the electrolyte before and after the electrochemical test was carried out using a S4 TSTAR, Bruker, equipped with a 17.5 keV Molybdenum primary excitation source (Figure S19).

## Supporting Information Available

Supplementary SEM and AFM images, XPS characterization, Faradaic efficiency, two-electrode test, EIS, SEC data, and TXRF (PDF)
Video showing the visual increase in the electrode area (MP4)
