# Supplementary material for: Highly Durable Nanoporous Cu2–xS Films for Efficient Hydrogen Evolution Electrocatalysis under Mild pH Conditions
Source: ACS Catal. 2023 Jul 26;13(15):10457–67. doi: 10.1021/acscatal.3c01673 (PMC10411506; doi:10.1021/acscatal.3c01673)
Supplement: Supplementary file 1 — cs3c01673_si_001.pdf [file cs3c01673_si_001.pdf]

Supporting information for

Highly Durable Nanoporous $Cu_{2-x}S$ Films for Efficient Hydrogen Evolution Electrocatalysis at Mild pH Conditions

Roser Fernández-Climent,[†] *Jesús Redondo,*[‖,¶] *Miguel García-Tecedor,*[†,β] *Maria Chiara Spadaro,*[‡] *Junnan Li,*[⊥] *Daniel Chartrand,*[⊥] *Frederik Schiller,*[π,θ] *Jhon Pazos,*[§] *Mikel F. Hurtado,*[§,Δ] *Victor de la Peña O'Shea,*[β] *Nikolay Kornienko*[⊥]*, Jordi Arbiol,*[‡,£] *Sara Barja,*[‖,θ,φ] ** Camilo A. Mesa*[†,§]** and Sixto Giménez*[†]***

† Institute of Advanced Materials (INAM), Universitat Jaume I, Av. de Vicente Sos Baynat, s/n, 12006, Castelló, Spain.

‖ Department of Polymers and Advanced Materials, Centro de Física de Materiales, University of the Basque Country UPV/EHU, 20018, San Sebastián, Spain

¶ Department of Surface and Plasma Science, Faculty of Mathematics and Physics, Charles University, 180 00, Prague 8, Czech Republic.

β Photoactivated Processes Unit, IMDEA Energy Institute, Parque Tecnológico de Móstoles, Avda. Ramón de la Sagra 3, 28935, Móstoles, Madrid, Spain.

‡ Catalan Institute of Nanoscience and Nanotechnology (ICN2) and BIST Campus UAB, Ballaterra, 08193, Barcelona, Catalonia, Spain.

⊥ Department of Chemistry, Université de Montréal, 1375 Ave. Thérèse-Lavoie-Roux, Montréal, QC H2V 0B3, Canada

π Centro de Física de Materiales and Material Physics Center CSIC/UPV-EHU, Manuel Lardizabal 5, 20018, San Sebastián, Spain.

θ Donostia International Physics Center, 20018, San Sebastián, Spain

φ IKERBASQUE, Basque Foundation for Science, 48009 Bilbao, Spain

§ Research Cluster on Converging Sciences and Technologies (NBIC), Departamento de Ingeniería Electrónica, Universidad Central, Calle 5 No 21-38, Bogotá, 110311, Colombia.

Δ Materials Chemistry Area, Civil Engineering Deparment, Corporación Tecnológica Minuto de Dios, Calle 80, Main Sede Bogotá, Colombia. – Nanotechnology Applications Area, Environmental Engineering Department, Universidad Militar Nueva Granada, Km 2 via Cajicá, Zipaquirá Colombia.

£ ICREA, Pg. Lluís Companys 23, 08010 Barcelona, Catalonia, Spain

*Corresponding authors. E-mail: sara.barja@ehu.eus, cmesa@uji.es, sjulia@uji.es

Table S1. Comparison of the HER performance of the Cu_2S electrocatalyst presented in this work with other Cu_2S and Cu-based materials reported in the literature. Note that the best performing materials, corresponding to references 9, 11 and 16 possess non-stoichiometric composition or highly defective polymorphs.

Materials	Solution	Overpotentials(ŋ)	Stability	Tafel slope	Ref
Cu_2S	0.1 M $KHCO_3$	132 mV @10 mA cm^{-2}	30 days	199 mV dec^{-1}	This work
γ-Cu_2S	1 M PBS	190 mV @10 mA cm^{-2}	10 h	98.8 mV dec^{-1}	1
CuS	0.5 M PBS	550 mV @10 mA cm^{-2}	-	149 mV dec^{-1}	2
Cu_2S@Cu	1 M KOH	316 mV @10 mA cm^{-2}	10 h	76 mV dec^{-1}	3
V-Cu_2S- NW	1 M KOH	188 mV @10 mA cm^{-2}	20 h	82.6 mV dec^{-1}	4
Cu_2S	1 M KOH	330 mV @10 mA cm^{-2}	24 h	106.93 mV dec^{-1}	5
Cu_2Se	0.5 M H_2SO_4	320 mV @10 mA cm^{-2}	-	79 mV dec^{-1}	6
Cu_2S NRs	1 M KOH	352 mV @ 50 mA cm^{-2}	-	141 mV dec^{-1}	7
Cu_2S NRs@CoS	1 M KOH	235 mV @ 50 mA cm^{-2}	36 h	121 mV dec^{-1}	7
Cu_2S-$Co(OH)_2$ NT	1 M KOH	241 mV @ 50 mA cm^{-2}	48 h	92 mV dec^{-1}	8
Cu_2S/MoS_2/CF	1 M KOH	91 mV @ 10 mA cm^{-2}	4 h	41 mV dec^{-1}	9
Cu_2S- Cu_3P	1 M KOH	158 mV @10 mA cm^{-2}	75 h	45 mV dec^{-1}	10
$Cu/Cu_2O/Cu_2S$ NT	0.5 M H_2SO_4	86 @10 mA cm-2	10 h	107 mV dec^{-1}	11
Cu_2S	0.5 M H_2SO_4	312 mV @ 10 mA cm^{-2}	24 h	49.79 mV dec^{-1}	5
MoS_2/CuS	0.5 M H_2SO_4	290 mV @ 10 mA cm^{-2}	6.6 h	63 mV dec^{-1}	12
Cu_2S/CF<br><br>Mo-Cu_2S/CF	1 M KOH	277 mV @ 10 mA cm^{-2}<br><br>18 mV @ 10 mA cm^{-2}	 <br>20 h	420 mV dec^{-1}<br><br>171 mV dec^{-1}	13
Cu_2S	Seawater	445 mV @ 10 mA cm^{-2}	10 h	136.1 mV dec^{-1}	14
Cu_2S	1 M KOH seawater	289 mV @ 100 mA cm^{-2}	12 h	128 mV dec^{-1}	15
$Cu_xS/MoS_2/TiO_2$	0.1 M H_2SO_4	~45 mV @ 10 mA cm^{-2}	10 days	~39 mV dec^{-1}	16

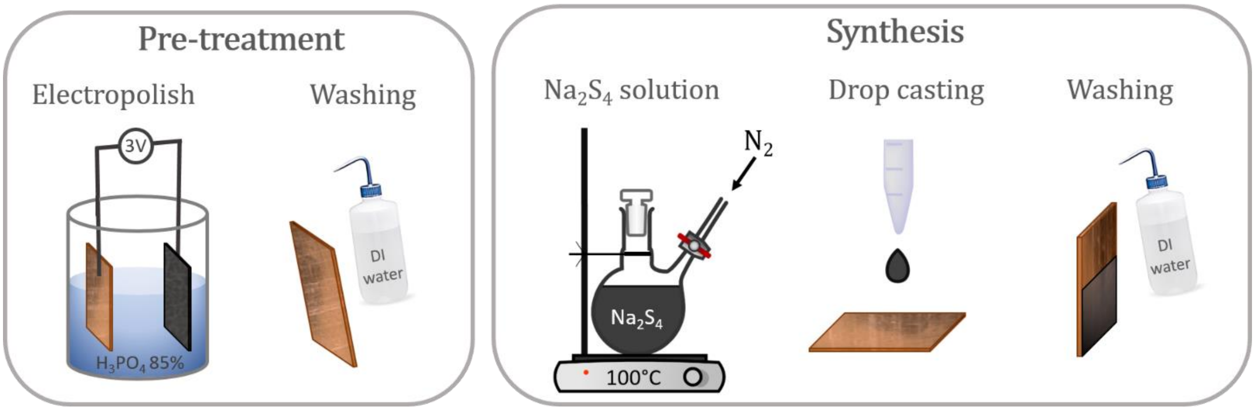

Figure S1. Schematic diagram illustrating the facile fabrication steps of $Cu_{2-x}S$ electrodes.

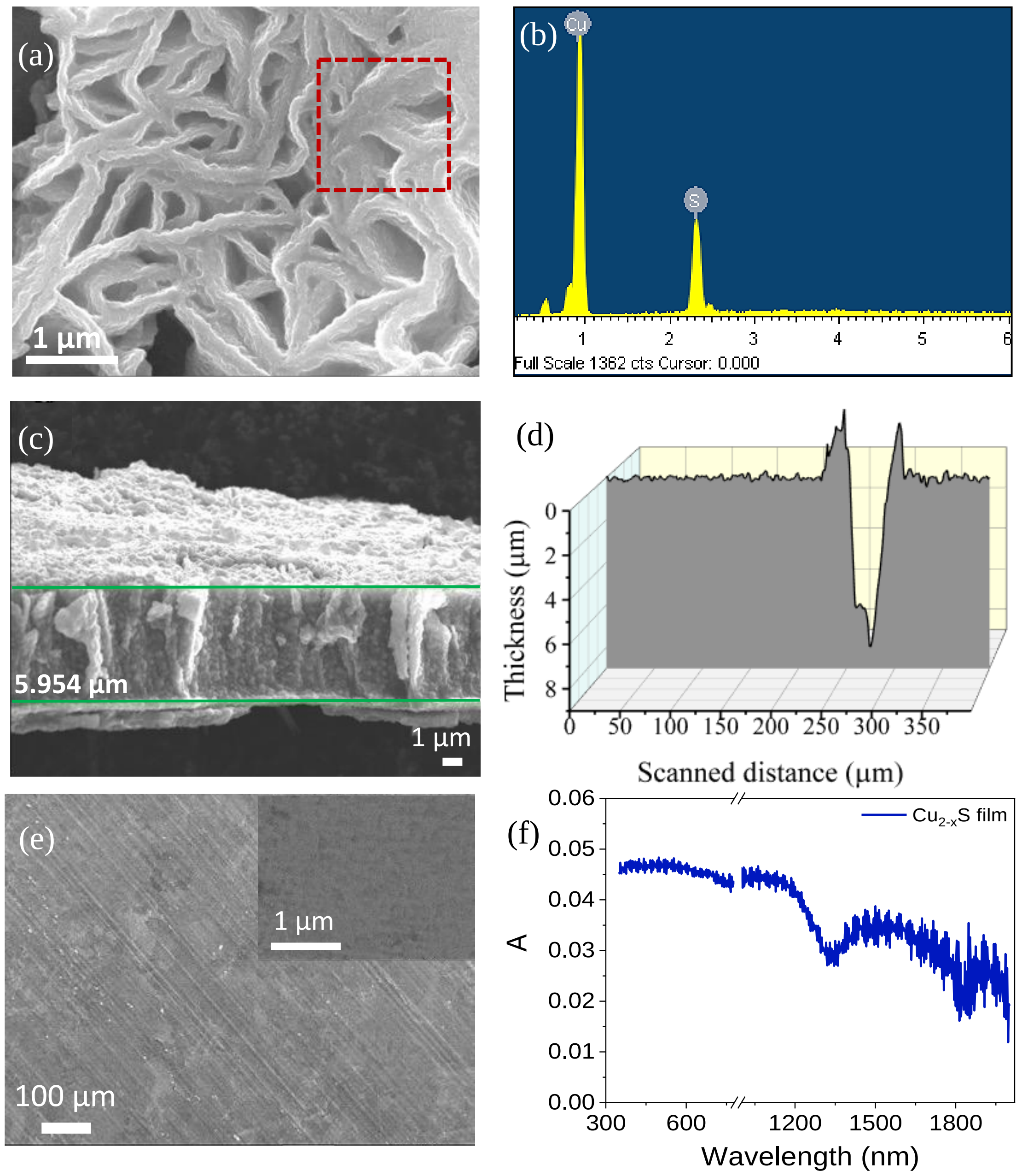

Figure S2. (a) SEM micrographs of the as-synthesized $Cu_{2-x}S$ porous films, (b) EDS spectrum of the red area in (a). (c) SEM Cross-sectional image showing the thickness of $Cu_{2-x}S$ around 6 µm. (d) Profilometry of a scratch on the surface of the $Cu_{2-x}S$ electrocatalyst. The thickness of the film is in average 6.15 µm. Note that the deeper data is due to a scratch on the copper substrate. (e) SEM image of the electropolished Cu substrate at two different magnifications. (f) UV-Vis-NIR absorption spectrum of the pristine $Cu_{2-x}S$ films, extracted from diffuse reflectance measurements. The NIR band centered ~1600 nm is tentatively assigned to localized surface plasmon resonance caused by the Cu deficiency as observed in other $Cu_{2-x}S$ electrodes.[17-18]

Table S2. EDS quantification of the $Cu_{2-x}S$ electrodes (spectrum from Figure S1c).

Element	Weight [%]	Atomic [%]
S (K line)	20.72	34.12
Cu (L line)	79.28	65.88
Totals	100.00	100.00

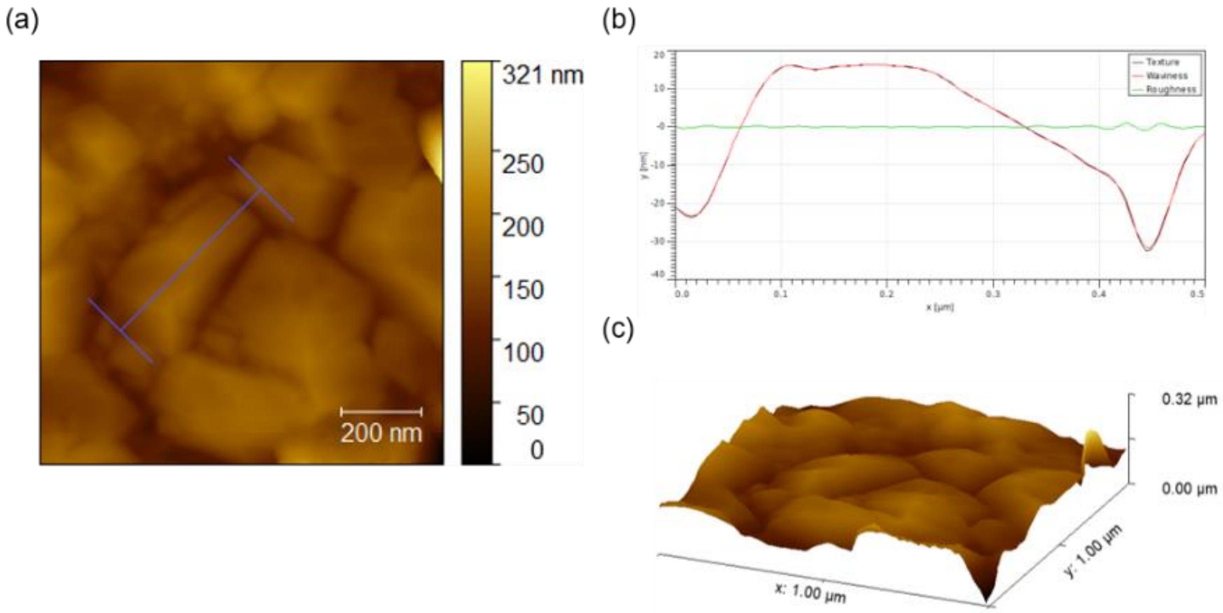

Figure S3. AFM analysis of the $Cu_{2-x}S$ electrodes. (a) Top view topography, (b) roughness analysis on a nano-hexagon structure selected in (a) exhibiting lateral faces of ~200-400 nm length and ~200 nm width and (c) 3D projection of the $Cu_{2-x}S$ electrode from the top view topography.

Deeper in the porous structure walls, crystalline hexagonal-shaped nanoparticles were observed and further characterized by atomic force microscopy (AFM) topography (**Figure S3**). These hexagonal formations were previously reported.[5, 19].

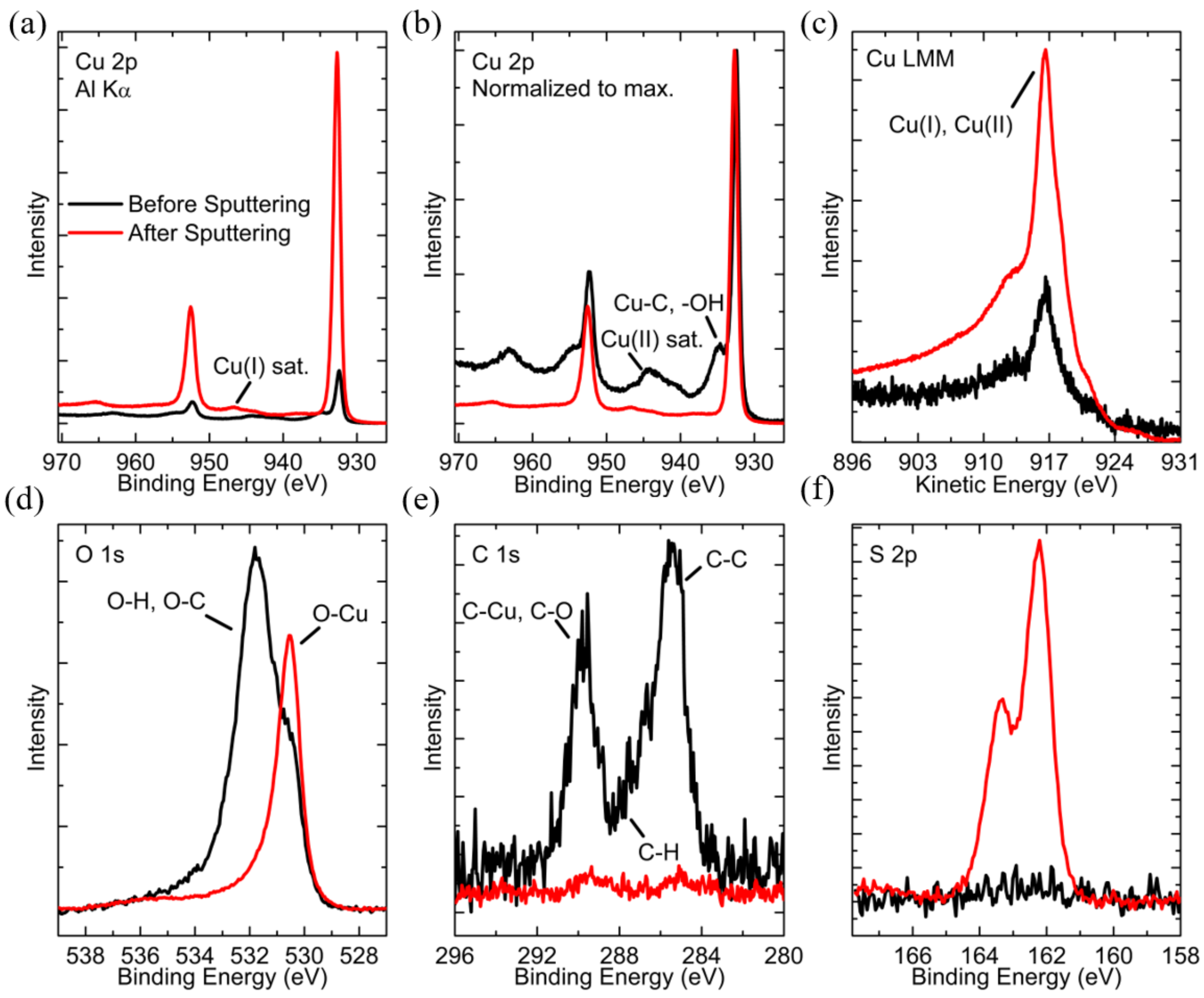

Figure S4. XPS analysis of the $Cu_{2-x}S$ as-synthetized electrodes before (black) and after (red) Ar^+ cleaning. (a) Cu 2p spectra, (b) Normalized Cu 2p spectra, (c) Cu Auger spectra, (d) O 1s spectra, (e) C 1s spectra and (f) S 2p spectra.

Figure S4 shows the comparison of the chemical state of the as-synthesize $Cu_{2-x}S$ electrodes before and after sputtering in UHV. The non-normalized Cu2p spectra, **Figure S4a**, show an 8x signal increase after the sputtering. Cu(II) satellite and elemental fingerprints[20-21] can only be identified before the sputtering in the normalized Cu2p spectra, **Figure S4b**. The Cu LMM auger peak, **Figure S4c** show no signatures of metallic Cu. Here, unambiguous identification of Cu(I) and Cu(II) states cannot be provided since they overlap in Cu_2S/CuS and Cu_2O/CuO materials.[20] A large decrease in the C

components of the O 1s and C 1s spectra, **Figures S4d** and **e** is seen after sputtering. The signal from sulfur, **Figure S4f** is only detectable after sputtering. All in all, the identification of the $Cu_{2-x}S$ electrode is only viable after sputtering. $Cu_{2-x}S$ is known to readily oxidize upon air exposure.[22] Note that a certain amount of Cu_2O is still present in the sample after sputtering, probably due to an inhomogeneous oxidation in air.

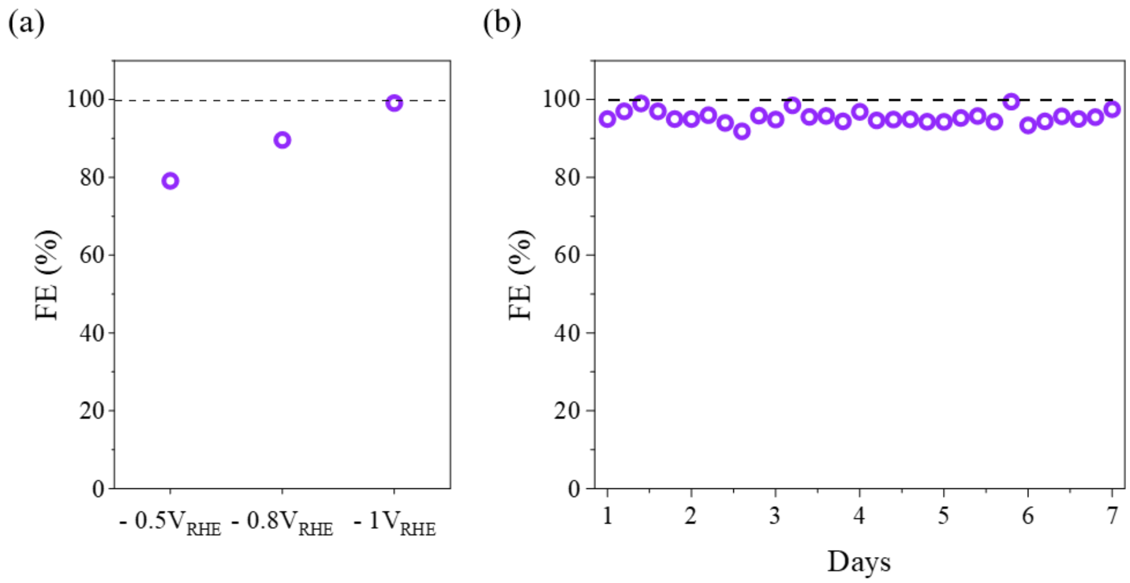

Figure S5. Hydrogen evolution measurements by gas chromatography. a) Faradaic efficiency extracted at three different applied potentials, -0.5 V vs RHE, -0.8 V vs RHE and -1V vs RHE and b) Faradaic efficiency for several days of continuous operation at -1 V vs RHE. The Faradaic Efficiencies lower that 100% at -0.5 and -0.8 V vs RHE are tentatively attributed to the competing CO_2 reduction from $KHCO_3$ electrolyte and experimental errors.

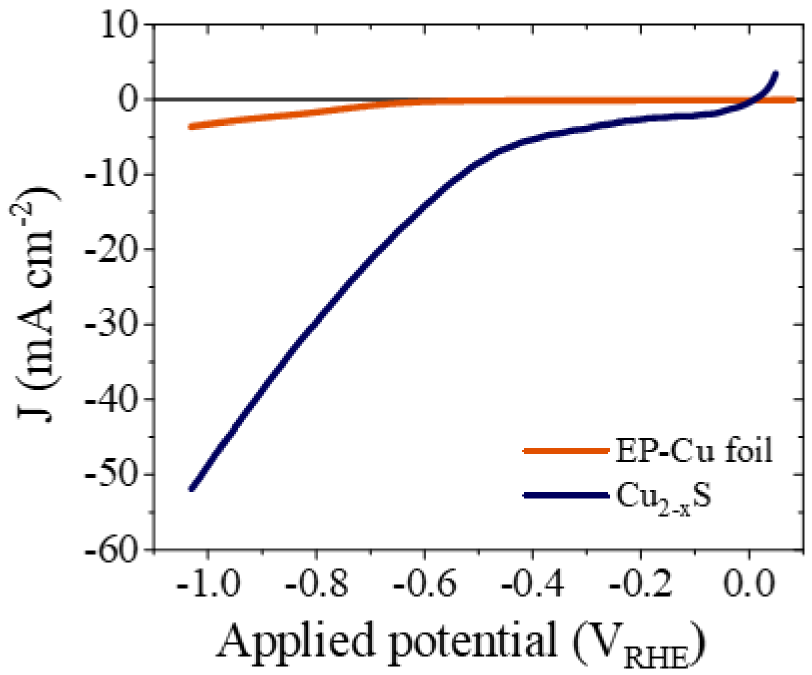

Figure S6. Linear sweep voltammetries comparison between the electropolished copper substrate (EP-Cu foil) and the copper sulfide electrocatalyst.

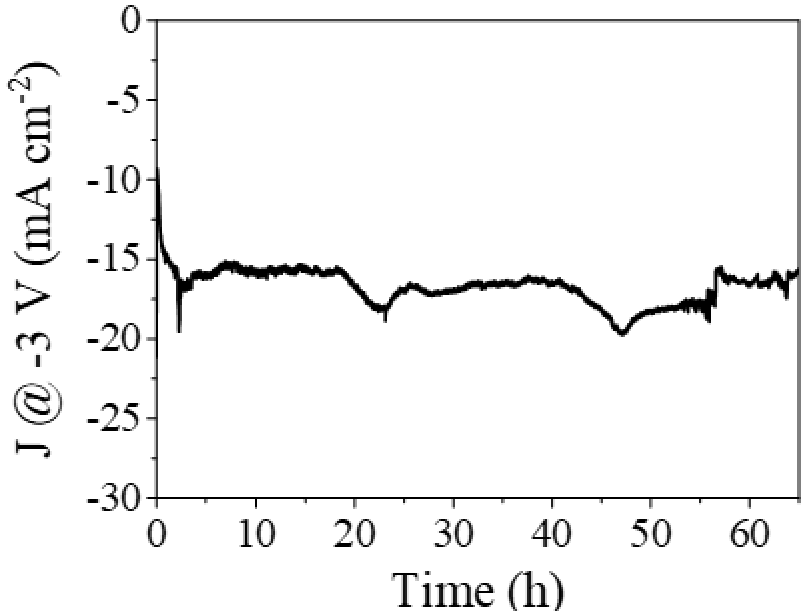

Figure S7. Chronoamperometric measurement in a two-electrode configuration in 0.1 M $KHCO_3$ electrolyte. The cyclic oscillations in J are mostly related to temperature variations at the lab during the measurements.

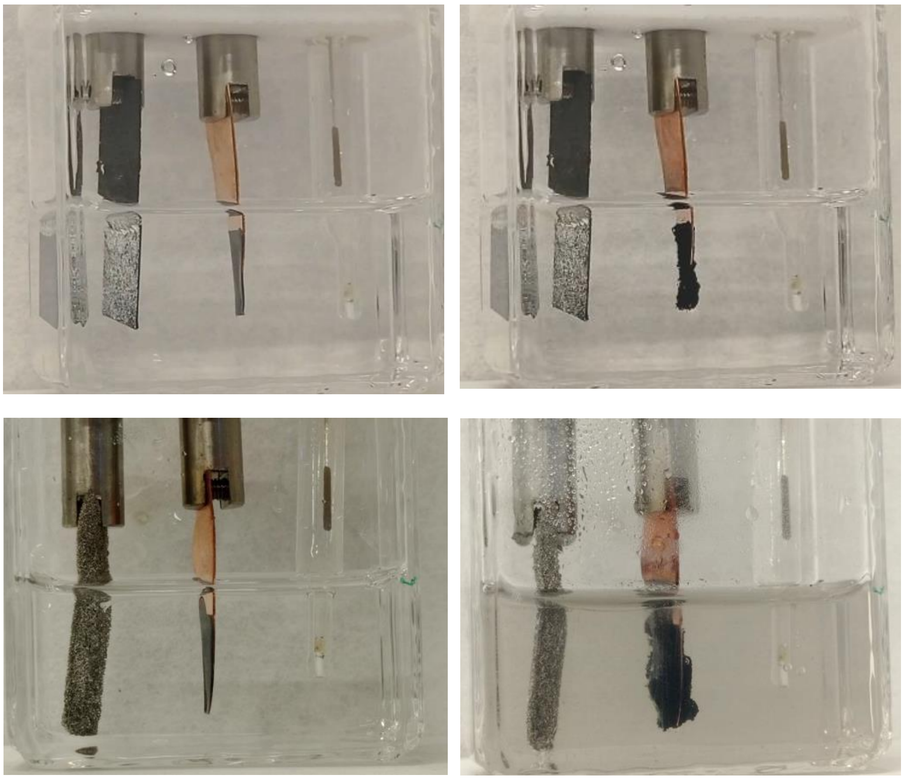

Figure S8. Pictures of the electrode (a) before and (b) after the electrochemistry using carbon sheet as a counter electrode an (c) before and (d) after the electrochemistry using a Ni foam as a counter electrode.

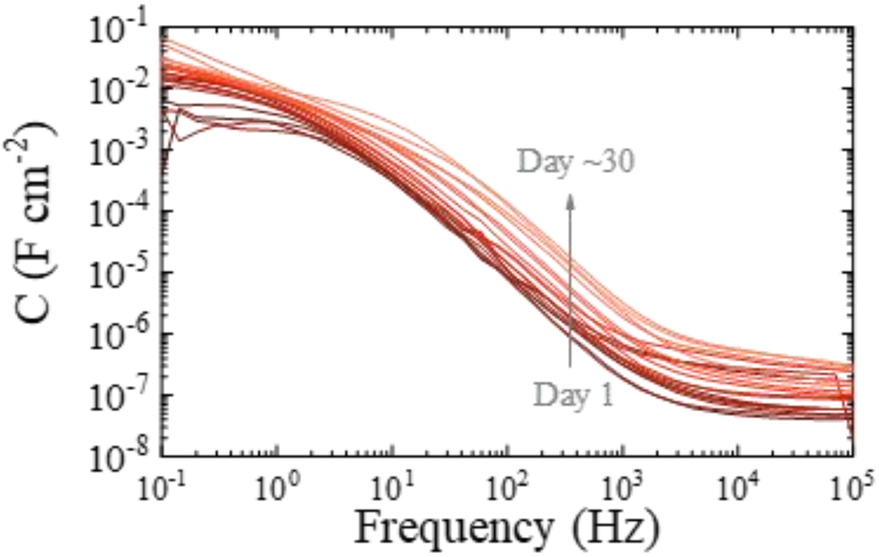

Figure S9. Real part of the complex capacitance measured as a function of frequency (Bode plots) of our $Cu_{2-x}S$ electrodes a function of operation time measured at -0.1 V *vs* RHE (non-faradaic region). The C_{dl} values were taken at ~5 Hz.

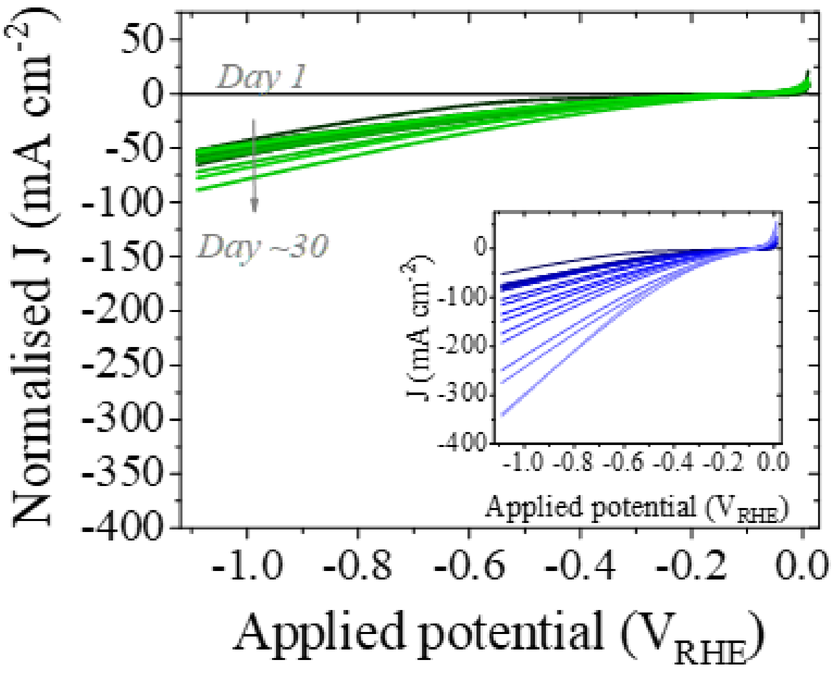

Figure S10. Normalized linear sweep voltammograms (LSV) by the r_{ECSA} values from **Figure 3c** of the same $Cu_{2-x}S$ electrode as a function of operation time between the day 1, i.e., freshly synthesized catalyst (darker blue) and after 28 days (lighter blue) of continuous operation. LSVs from **Figure 3b** are displayed in the inset for reference. The LSV were measured at 20 mV s^{-1} in 0.1 M $KHCO_3$.

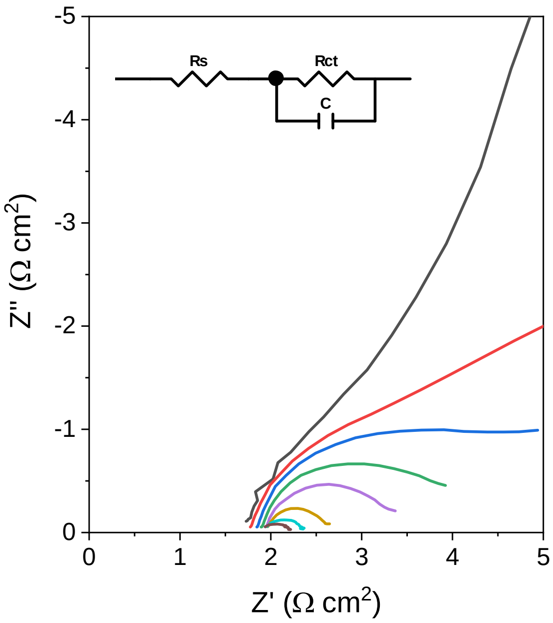

Figure S11. Nyquist plots. Real versus imaginary part of the complex impedance for $Cu_{2-x}S$ electrocatalyst at different applied voltages and the employed Randles' equivalent circuit for EIS analysis.

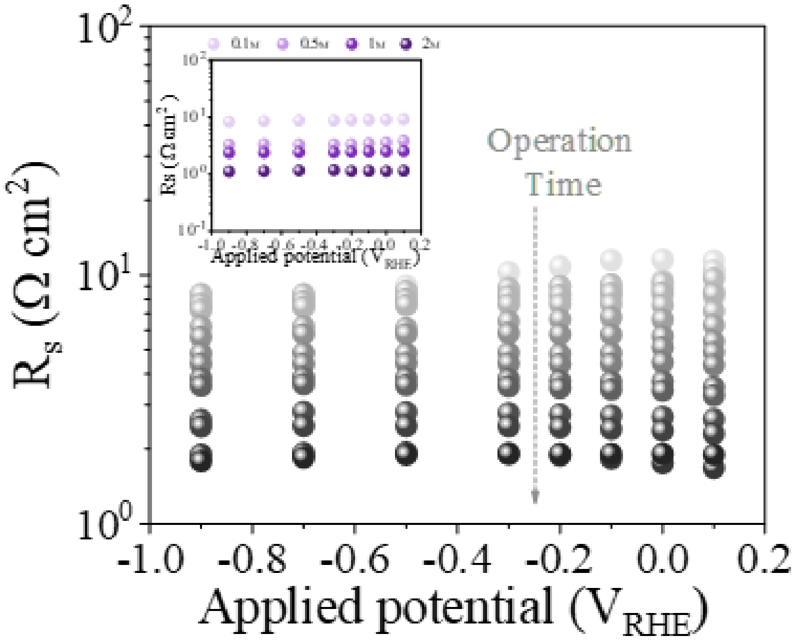

Figure S12. R_s values of the $Cu_{2-x}S$ catalysts extracted from electrochemical impedance spectroscopy (EIS) analysis as a function of operation time (indicated by the grey arrow), inset: R_s values of the Cu_2S catalysts measured as a function of concentration of $KHCO_3$ electrolyte. A 20-fold increase of the $KHCO_3$ concentration results in a decrease of ~1 order of magnitude in the R_s, thus, the observed ohmic drop decrease can be attributed to an increase in ionic concentration in the electrolyte. *Rs* can aid understanding the difference between the 8-fold increase in *J*, compared to the 6.5-fold increase in *ECSA* shown in **Figure 3c**. However, this is out of the scope if this paper and is being subject of further analysis.

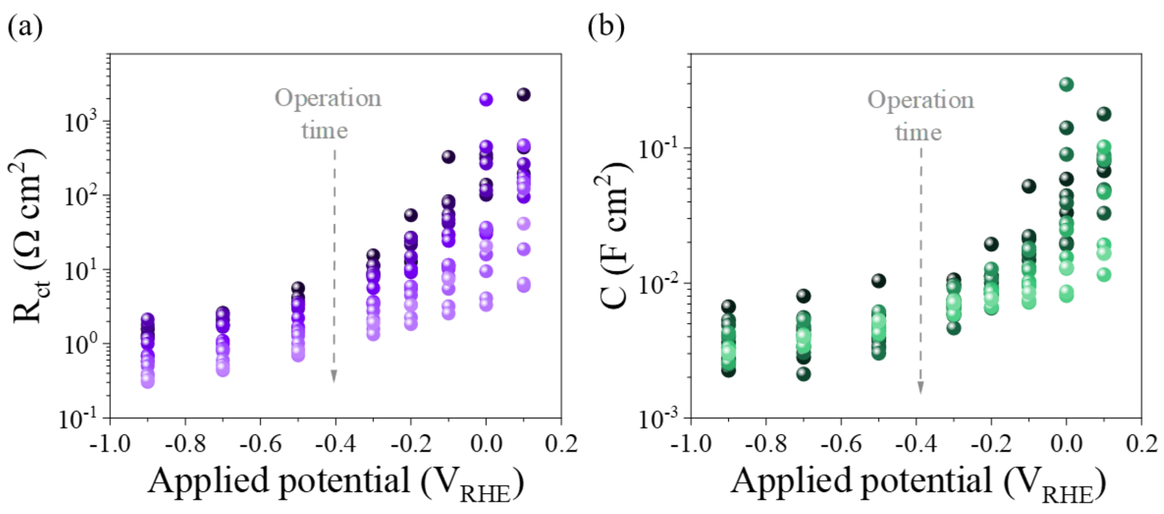

Figure S13. Different extracted parameters from EIS measurements: a) Charge transfer resistances (R_{ct}) b) Capacitances values as a function of operation time from the $Cu_{2-x}S$ electrocatalysts.

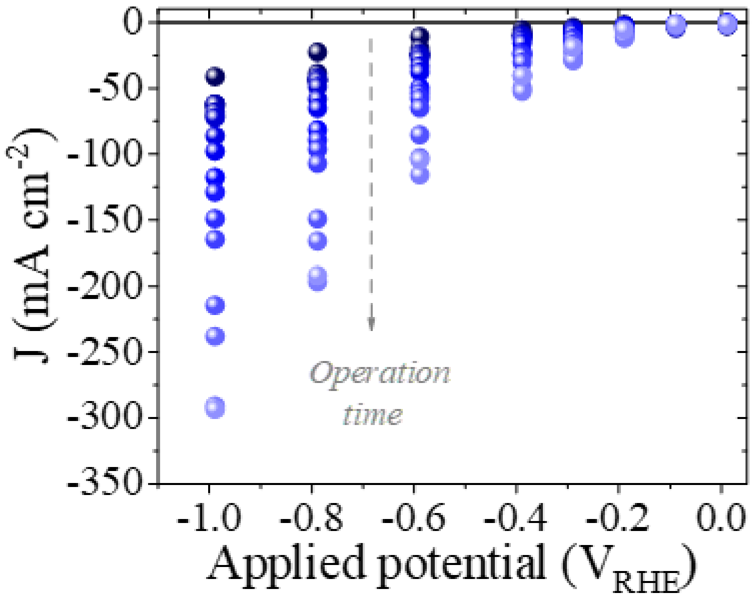

Figure S14. Steady-state *LSVs* of the $Cu_{2-x}S$ electrode as a function of operation time between the day 1, i.e., freshly synthesized catalyst (darker blue) and after 28 days (lighter blue) of continuous operation (indicated by the grey arrow). Every data point corresponds

to the average current of the last 60 s of a 5-minute chronoamperometric measurement at every measured potential.

SEC supplementary analysis

To collect more information on the interaction between the $Cu_{2-x}S$ electrode and the electrolyte, complementary experiments were performed in inert electrolyte and using Cu foil as reference under a strong negative bias. A UV-Vis-NIR spectrum was measured while applying a catalytic potential of -1 V_{NHE} in dry 0.1 M TBAP in acetonitrile to study the electrocatalyst under potential without hydrogen generation taking place. The corresponding spectrum (dark red spectrum) in **Figure S13**, exhibits an almost flat absorption. When few drops of DI water were added to the 0.1 M TBAP in acetonitrile (orange spectrum), and -1 V_{NHE} was applied, a wide absorption appears at ~1500 nm as a consequence of the presence of free water. Upon comparing these two spectra with the one obtained in 0.1 M $KHCO_3$ under -1 V_{RHE} when the $Cu_{2-x}S$ is evolving hydrogen, blue spectrum in **Figure S13**, two new absorption peaks appear ~1080nm and at ~1270 nm which can be tentatively attibuted to the protons bound to the electrocatalyst. Optical signals ~550 and 1370 nm appears in all spectra, including when no reaction is taking place (dark red spectrum, inert electrrolyte). Both signals are assigned to optical absorptions from the $Cu_{2-x}S$ electrode in agreement with previous reports.[17, 23] In addition to this, the Cu-foil blank exhibits an additional absorption peak ~600-650 nm in **Figure 5a** upon strong bias application (-1.1 V vs RHE) which is not present in the $Cu_{2-x}S$ spectra at similar bias. This indicates that most likely the observed HER activity comes from the our $Cu_{2-x}S$ electrodes rather than the substrate.

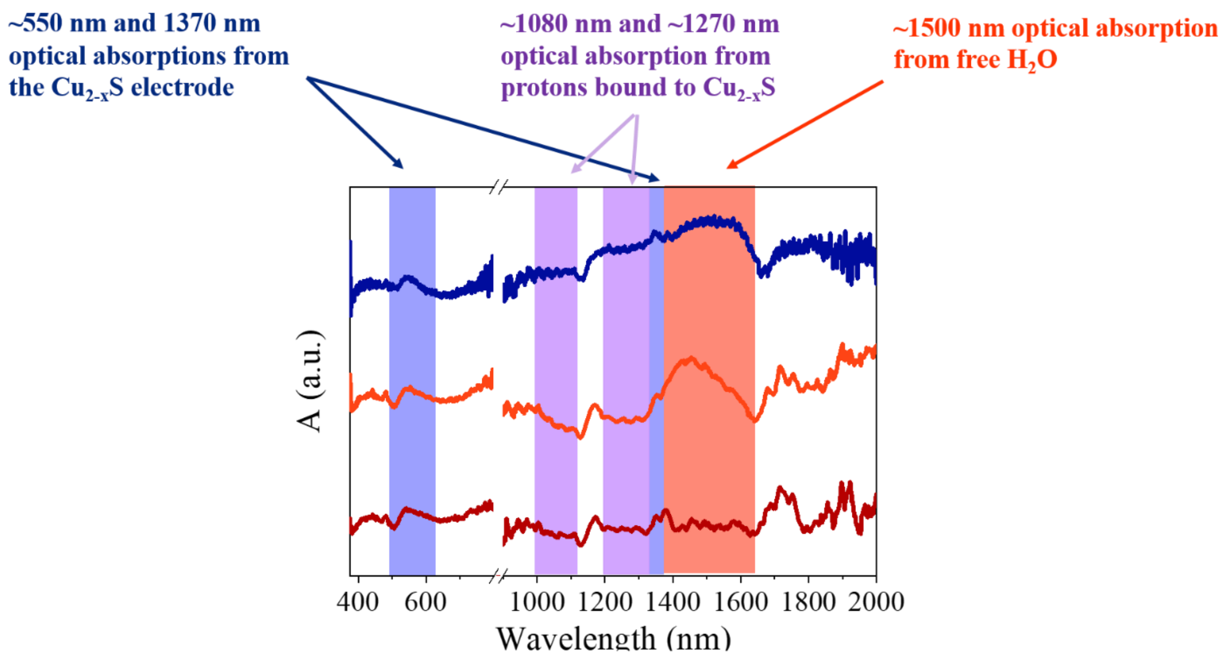

Figure S15. UV-Vis-NIR absorption spectra of $Cu_{2-x}S$ electrode in 0.1 M $KHCO_3$ (dark blue), 0.1 M TBAP in acetonitrile and water (orange) and 0.1 M TBAP in acetonitrile (dark red) while applying -1 V vs RHE.

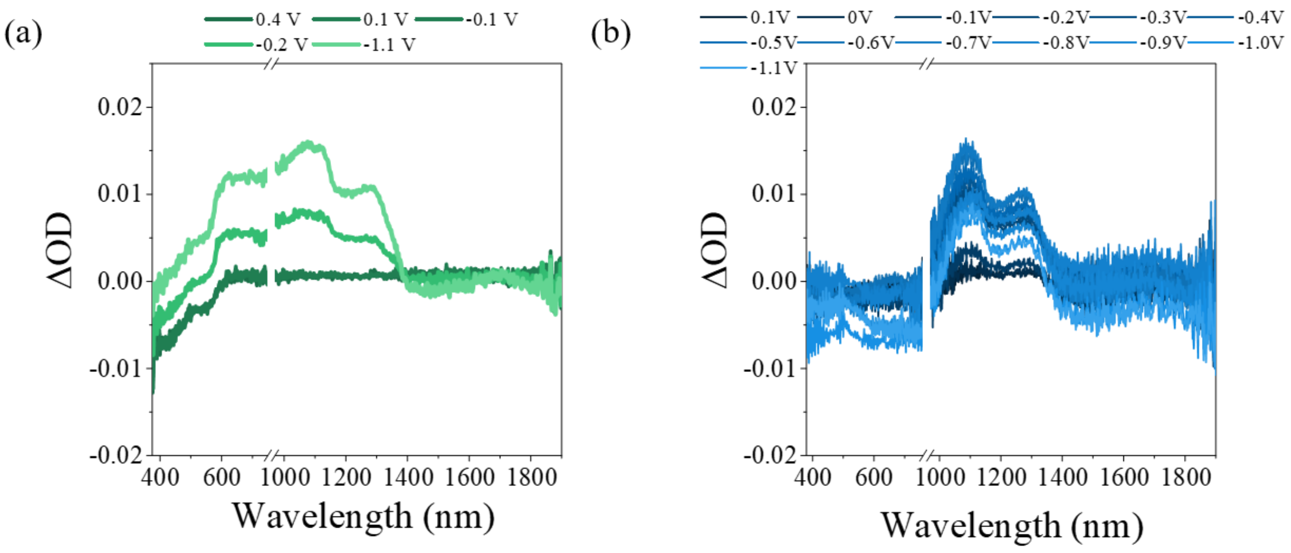

Figure S16. Differential spectra of the (a) reference Cu foil and (b) $Cu_{2-x}S$ and reference electrodes as a function of applied potential.

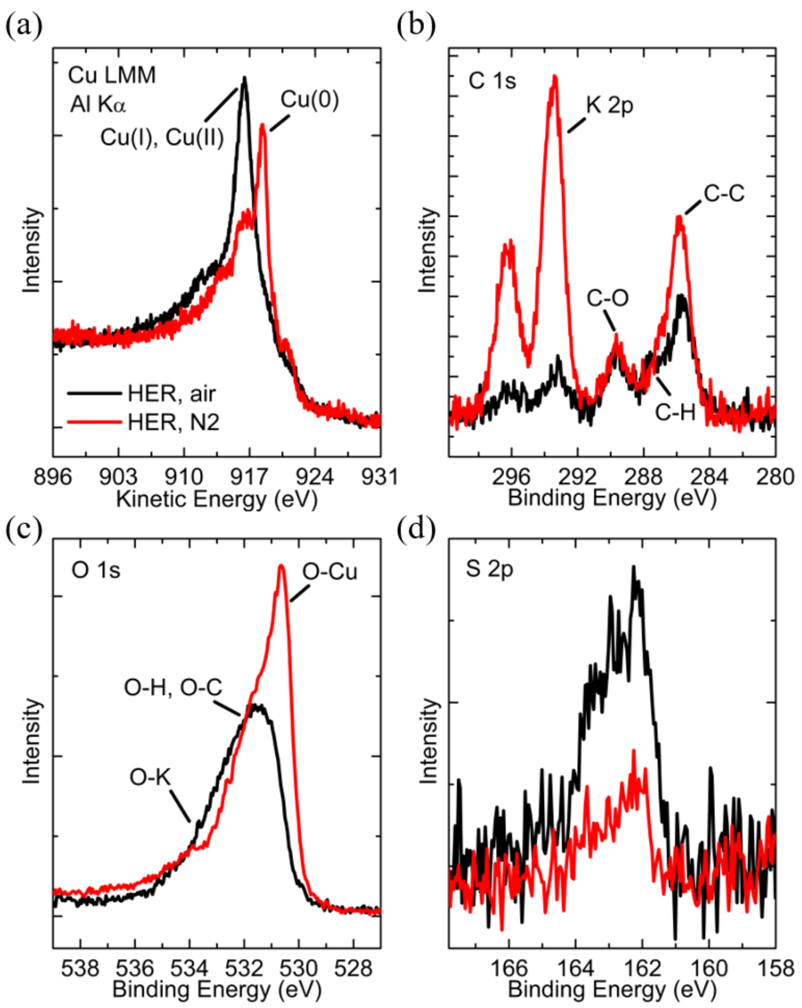

Figure S17. XPS analysis of the $Cu_{2-x}S$ electrodes after the electrochemical measurements (a) Cu Auger spectra, (b) C 1s spectra, (c) O 1s spectra and (d) 2p spectra when transferred under air (black) and under N_2 atmospheres (red).

Figure S17 shows a comparison of HER characterization and transport to the UHV chamber performed under Air and N_2 atmospheres. These samples were not rinsed after HER nor sputtered in UHV. The Cu LMM spectra shows no signal of metallic Cu in the air-sample. Only the samples produced and transported in N_2 show metallic Cu. Different levels of K from the electrolyte solution are found on these samples. Note that the lower amount of S in the N_2-sample is the result of a higher signal attenuation from the higher K and C amounts.

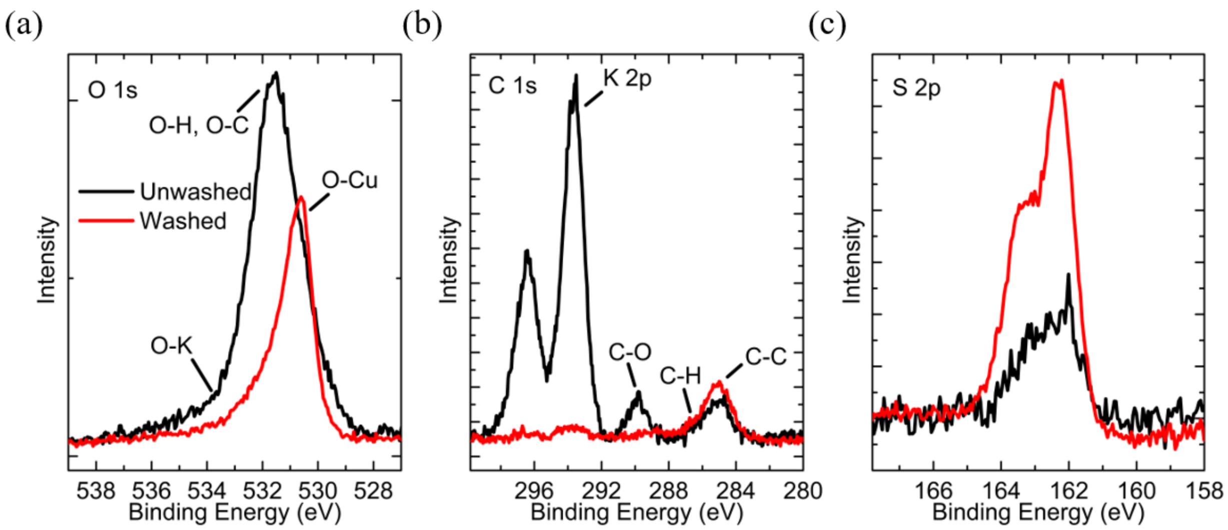

Figure S18 XPS analysis of the $Cu_{2-x}S$ electrodes after the electrochemical measurements with and without washing with water.

Figure S18 shows the results of MiliQ-water rinsing of samples after HER. The samples here were produced and transported under N2 atmosphere. A large amount of C and K from the electrolyte is detected. The S signal is largely attenuated by the electrolyte residues. Water-rinsing of $Cu_{2-x}S$ electrodes is known to remove these residues without altering their chemical configuration.[24]

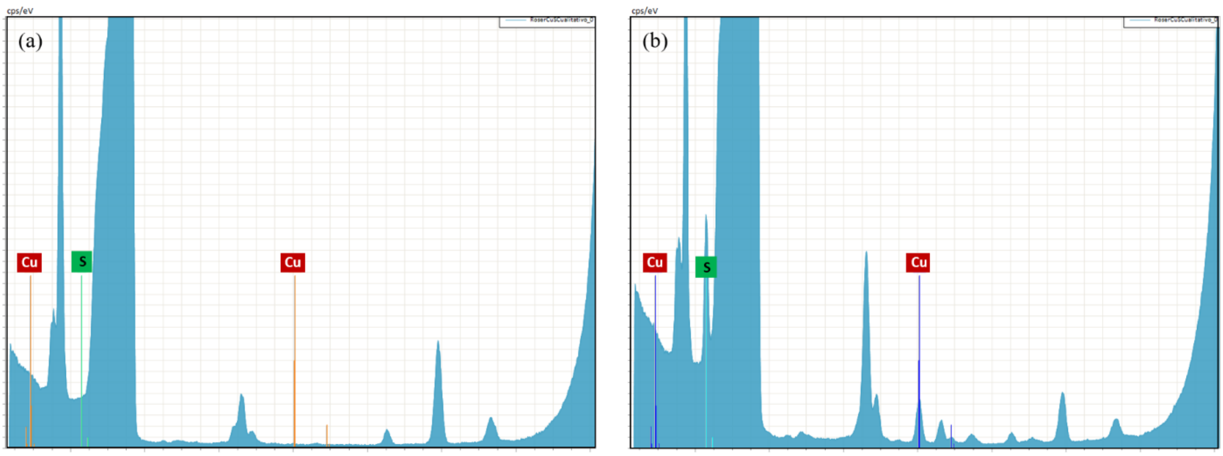

Figure S19. Total reflection X-rays fluorescence (TXRF) analysis of the electrolyte (a) before and (b) after the electrochemical test.

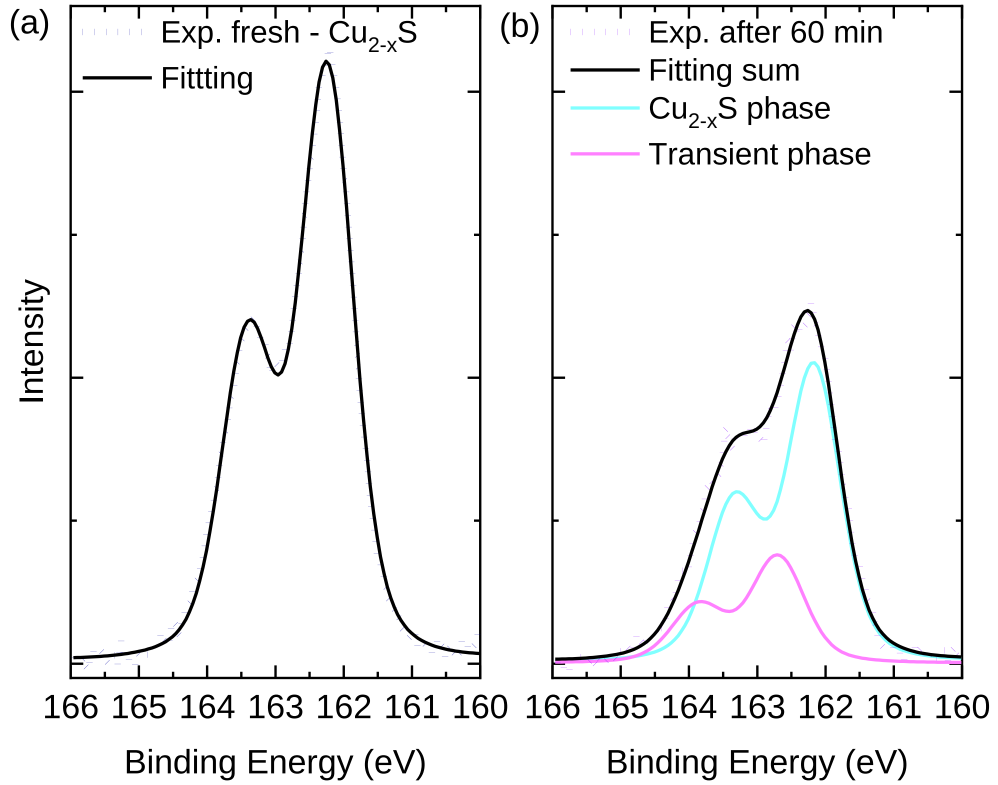

Figure S20: Deconvoluted analysis of the spectral features in the S 2p spectrum of the (a) fresh $Cu_{2-x}S$ electrode, and (b) after 60 min of operation. The S 2p region of the fresh $Cu_{2-x}S$ represents a single doublet function at 162.2 eV with a spin-orbit splitting of 1.17 eV and a 0.53 intensity ratio. The S 2p spectrum of the electrode after 60 min of electrochemical treatment reveals an important reduction of its initial intensity and present a second chemical component at higher binding energies, with the described doublet at 162.7 eV. This component adds up to approximately 38% of the total S signal.

REFERENCES

1. Fan, M.; Gao, R.; Zou, Y.-C.; Wang, D.; Bai, N.; Li, G.-D.; Zou, X., An efficient nanostructured copper (I) sulfide-based hydrogen evolution electrocatalyst at neutral pH. *Electrochimica Acta* **2016,** *215*, 366-373.

2. Li, M.; Qian, Y.; Du, J.; Wu, H.; Zhang, L.; Li, G.; Li, K.; Wang, W.; Kang, D. J., CuS nanosheets decorated with CoS_2 nanoparticles as an efficient electrocatalyst for enhanced hydrogen evolution at all pH values. *ACS Sustainable Chemistry & Engineering* **2019,** *7* (16), 14016-14022.
3. Ma, B.; Yang, Z.; Yuan, Z.; Chen, Y., Effective surface roughening of three-dimensional copper foam via sulfurization treatment as a bifunctional electrocatalyst for water splitting. *International Journal of Hydrogen Energy* **2019,** *44* (3), 1620-1626.
4. Yang, D.; Cao, L.; Huang, J.; Liu, Q.; Li, G.; He, D.; Wang, J.; Feng, L., Vanadium-doped hierarchical Cu_2S nanowall arrays assembled by nanowires on copper foam as an efficient electrocatalyst for hydrogen evolution reaction. *Scripta Materialia* **2021,** *196*, 113756.
5. Bhat, K. S.; Nagaraja, H. S., Hydrogen evolution reaction at extreme pH conditions of copper sulfide micro-hexagons. *Journal of Science: Advanced Materials and Devices* **2020,** *5* (3), 361-367.
6. Durairaj, A.; Sakthivel, T.; Ramanathan, S.; Obadiah, A.; Vasanthkumar, S., Hierarchical Cu_2Se nanostructures film for peroxymonosulfate activation and electrocatalytic hydrogen evolution. *Journal of the Taiwan Institute of Chemical Engineers* **2019,** *99*, 66-73.
7. Zhou, Q.; Li, T.-T.; Wang, J.; Guo, F.; Zheng, Y.-Q., Hierarchical Cu_2S NRs@ CoS core-shell structure and its derivative towards synergistic electrocatalytic water splitting. *Electrochimica Acta* **2019,** *296*, 1035-1041.
8. Wang, D.; Li, J.; Zhao, Y.; Xu, H.; Zhao, J., Bifunctional Cu_2S–Co $(OH)_2$ nanotube array/Cu foam electrocatalyst for overall water splitting. *Electrochimica Acta* **2019,** *316*, 8-18.
9. Wang, X.; Wang, J.; Zhang, X.; Tian, Q.; Liu, M.; Cai, N.; Xue, Y.; Chen, W.; Li, W.; Yu, F., Nitrogen-Doped Cu_2S/MoS_2 Heterojunction Nanorod Arrays on Copper Foam for Efficient Hydrogen Evolution Reaction. *ChemCatChem* **2019,** *11* (4), 1354-1361.
10. Xu, F.; Lu, J.; Luo, L.; Yu, C.; Tang, Z.; Abbo, H. S.; Titinchi, S. J. J.; Zhu, J.; Kang Shen, P.; Yin, S., Cu2S-Cu3P nanowire arrays self-supported on copper foam as boosting electrocatalysts for hydrogen evolution. *Energy Technology* **2019,** *7* (4), 1800993.
11. Wei, Y.; He, W.; Sun, P.; Yin, J.; Deng, X.; Xu, X., Synthesis of hollow $Cu/Cu_2O/Cu_2S$ nanotubes for enhanced electrocatalytic hydrogen evolution. *Applied Surface Science* **2019,** *476*, 966-971.
12. Zhang, L.; Guo, Y.; Iqbal, A.; Li, B.; Gong, D.; Liu, W.; Iqbal, K.; Liu, W.; Qin, W., One-step synthesis of the 3D flower-like heterostructure MoS_2/CuS nanohybrid for electrocatalytic hydrogen evolution. *International Journal of Hydrogen Energy* **2018,** *43* (3), 1251-1260.
13. Xie, Y.; Huang, J.; Xu, R.; He, D.; Niu, M.; Li, X.; Xu, G.; Cao, L.; Feng, L., Mo-doped Cu_2S multilayer nanosheets grown in situ on copper foam for efficient hydrogen evolution reaction. *Molecules* **2022,** *27* (18), 5961.
14. Marimuthu, T.; Yuvakkumar, R.; Ravi, G.; Zheng, Y.; Bi, Z.; Xu, X.; Xu, G.; Velauthapillai, D., One-step fabrication of copper sulfide catalysts for HER in natural seawater and their bifunctional properties in freshwater splitting. *Fuel* **2022,** *322*, 124073.
15. Marimuthu, T.; Yuvakkumar, R.; Kumar, P. S.; Ravi, G.; Xu, X.; Velauthapillai, D.; Dai Viet, N. V., Cost effective and facile low temperature hydrothermal fabrication of Cu2S thin films for hydrogen evolution reaction in seawater splitting. *International Journal of Hydrogen Energy* **2022,** *47* (72), 30819-30829.

16. Bae, C.; Ho, T. A.; Kim, H.; Lee, S.; Lim, S.; Kim, M.; Yoo, H.; Montero-Moreno, J. M.; Park, J. H.; Shin, H., Bulk layered heterojunction as an efficient electrocatalyst for hydrogen evolution. *Science advances* **2017,** *3* (3), e1602215.
17. Van Der Stam, W.; Gudjonsdottir, S.; Evers, W. H.; Houtepen, A. J., Switching between plasmonic and fluorescent copper sulfide nanocrystals. *Journal of the American Chemical Society* **2017,** *139* (37), 13208-13217.
18. Luther, J. M.; Jain, P. K.; Ewers, T.; Alivisatos, A. P., Localized surface plasmon resonances arising from free carriers in doped quantum dots. *Nature materials* **2011,** *10* (5), 361-366.
19. Wu, Y.; Wadia, C.; Ma, W.; Sadtler, B.; Alivisatos, A. P., Synthesis and photovoltaic application of copper (I) sulfide nanocrystals. *Nano letters* **2008,** *8* (8), 2551-2555.
20. Biesinger, M. C.; Lau, L. W. M.; Gerson, A. R.; Smart, R. S. C., Resolving surface chemical states in XPS analysis of first row transition metals, oxides and hydroxides: Sc, Ti, V, Cu and Zn. *Applied surface science* **2010,** *257* (3), 887-898.
21. Biesinger, M. C., Advanced analysis of copper X-ray photoelectron spectra. *Surface and Interface Analysis* **2017,** *49* (13), 1325-1334.
22. Krylova, V.; Andrulevičius, M., Optical, XPS and XRD studies of semiconducting copper sulfide layers on a polyamide film. *International Journal of Photoenergy* **2009,** *2009*.
23. Xie, Y.; Riedinger, A.; Prato, M.; Casu, A.; Genovese, A.; Guardia, P.; Sottini, S.; Sangregorio, C.; Miszta, K.; Ghosh, S., Copper sulfide nanocrystals with tunable composition by reduction of covellite nanocrystals with Cu^+ ions. *Journal of the American Chemical Society* **2013,** *135* (46), 17630-17637.
24. Velásquez, P.; Leinen, D.; Pascual, J.; Ramos-Barrado, J. R.; Cordova, R.; Gómez, H.; Schrebler, R., XPS, SEM, EDX and EIS study of an electrochemically modified electrode surface of natural chalcocite (Cu_2S). *Journal of Electroanalytical Chemistry* **2001,** *510* (1-2), 20-28.
